# Biogenic Synthesis of Copper Nanoparticles: A Systematic Review of Their Features and Main Applications

**DOI:** 10.3390/molecules28124838

**Published:** 2023-06-18

**Authors:** Cristina M. Luque-Jacobo, Andrea L. Cespedes-Loayza, Talia S. Echegaray-Ugarte, Jacqueline L. Cruz-Loayza, Isemar Cruz, Júlio Cesar de Carvalho, Luis Daniel Goyzueta-Mamani

**Affiliations:** 1 Sustainable Innovative Biomaterials Department, Le Qara Research Center, Arequipa 04000, Peru; c.luque@leqara.com (C.M.L.-J.); t.echegaray@leqara.com (T.S.E.-U.); j.cruz@leqara.com (J.L.C.-L.); i.cruz@leqara.com (I.C.); 2 Bioprocess Engineering and Biotechnology Department, Federal University of Paraná—Polytechnic Center, Curitiba 81531-980, Brazil; jccarvalho@ufpr.br; 3 Vicerrectorado de Investigación, Universidad Católica de Santa María, Urb. San José s/n-Umacollo, Arequipa 04000, Peru

**Keywords:** copper nanoparticles, biogenic synthesis, systematic review, antibacterial, antioxidant, antitumoral, catalytic and removal activity

## Abstract

Nanotechnology is an innovative field of study that has made significant progress due to its potential versatility and wide range of applications, precisely because of the development of metal nanoparticles such as copper. Nanoparticles are bodies composed of a nanometric cluster of atoms (1–100 nm). Biogenic alternatives have replaced their chemical synthesis due to their environmental friendliness, dependability, sustainability, and low energy demand. This ecofriendly option has medical, pharmaceutical, food, and agricultural applications. When compared to their chemical counterparts, using biological agents, such as micro-organisms and plant extracts, as reducing and stabilizing agents has shown viability and acceptance. Therefore, it is a feasible alternative for rapid synthesis and scaling-up processes. Several research articles on the biogenic synthesis of copper nanoparticles have been published over the past decade. Still, none provided an organized, comprehensive overview of their properties and potential applications. Thus, this systematic review aims to assess research articles published over the past decade regarding the antioxidant, antitumor, antimicrobial, dye removal, and catalytic activities of biogenically synthesized copper nanoparticles using the scientific methodology of big data analytics. Plant extract and micro-organisms (bacteria and fungi) are addressed as biological agents. We intend to assist the scientific community in comprehending and locating helpful information for future research or application development.

## 1. Introduction

During the past two decades, extensive research has focused on synthesizing and utilizing nanoparticles, which play an essential role in multiple areas, including feed and pharmaceuticals, due to their unique properties [1]. Copper nanoparticles (CuNPs) have exhibited extraordinary properties due to their versatile activity, such as antitumor, antimicrobial, antioxidant, dye removal, and catalytic degradation. However, their traditional synthesis (chemical method) has issues, such as prolonged technical work and toxic raw materials, e.g., hydrazine, *N*-dimethylformamide, and sodium borohydride [2,3], which are potentially carcinogenic compounds [4]. In addition, the traditional chemical synthesis may present environmental risks, such as dispersion and exposure, to bioactive residues, with soil and water ecotoxicity.

According to Santhroshkumar et al. (2018) [1], green synthesis is most effective for reducing and stabilizing metal ions. Biological synthesis offers numerous benefits and methods, such as minimizing time consumption while providing non-toxicity and cost-effectiveness. In accordance with these statements, micro-organisms are capable of intracellular and cellular biosynthesis [5]. Most micro-organisms secrete extracellular enzymes with functional groups that have an affinity for metals, whereas some phytochemicals can perform cellular reduction, providing a natural coating that improves the NP biogenic stability. The cellular wall of fungi is composed of chitin and glucan, which can carry out the complexation of heavy metals that form metallic nanoparticles [6,7]. Despite the advantages of micro-organism synthesis, such as ease of handling, high growth capacity, and low environmental toxicity, the industrialization of nanoparticles is affected by the possibility of culture contamination, lengthy procedures, and a lack of control over their size [8].

On the other hand, biological synthesis utilizing natural plant extracts, such as leaves, fruits, stems, roots, flowers, seeds, or substances produced by plants, such as latex, have been used as reducing, stabilizing, and topping agents. The production of nanoparticles occurs through the interaction of the metal salt and organic compounds present in the extract, including proteins, amino acids, organic acids, vitamins, and secondary metabolites (bioactive compounds), such as flavonoids, alkaloids, polyphenols, terpenoids, heterocyclic compounds, and polysaccharides, which can act as binders [9]. In addition, these organic compounds can help control and form homogenous shapes, such as spherical, linear, and cubic, with an average size of 10 to 100 nm without the addition of synthetic polymers and surfactants [10]. Therefore, plant extracts can provide a more cost-effective method for nanoparticle synthesis than micro-organism synthesis [11], as evidenced by CuNPs.

CuNPs biologically synthesized using plant extracts are in high demand due to their wide variety of industrial uses and applications due to their innovative and tunable properties, such as high surface area, excellent conductivity, chemical reactivity, stability, and oxidation. As previously discussed, these nanoparticles can be utilized as antimicrobial and antifungal agents, where copper oxide NPs are more effective against Gram-positive and harmful bacteria such as *Escherichia coli*, *Staphylococcus aureus*, *Klebsiella pneumoniae*, *Salmonella typhi*, and *Bacillus subtilis* [12,13]. On the other hand, the antioxidant activity is associated with various bioreductive groups on the surface of the CuNPs [14]. Concerning antitumoral issues, CuNPs have been studied for their anticancer properties, which can be attained via multiple routes (reactive oxygen species-ROS generation, apoptosis, and autophagy, among others) that demonstrate the CuO nanoparticles’ anticancer ability toward cancer cell types; additionally, the type of plant used also affects anticancer response [15]. CuNPs synthesized from plant extracts are an environmentally friendly alternative for obtaining photocatalysts. Dye removal via catalytic degradation is another critical application of these nanoparticles, where various nanomaterials, such as transition metal oxides (i.e., CuO and Cu_2_O) are used. The precise reaction mechanism by which biogenic CuNPs and their oxides remove and degrade dyes is unknown. Nonetheless, numerous dyes can be converted into colorless or less pigmented reduced derivates because of dye degradation. 

The lack of an organized and comprehensive overview of the properties and potential applications of CuNPs demonstrates the need for the current study. However, a fragmented understanding of the synthesis of CuNPs and their primary applications leads to a variety of conclusions. Consequently, this study aims to assess the research articles published over the past decade on the different applications of biogenically synthesized CuNPs, including antioxidant, antitumor, antimicrobial, dye-scavenging, and catalytic activities.

## 2. Results and Discussions

**Figure 1:**
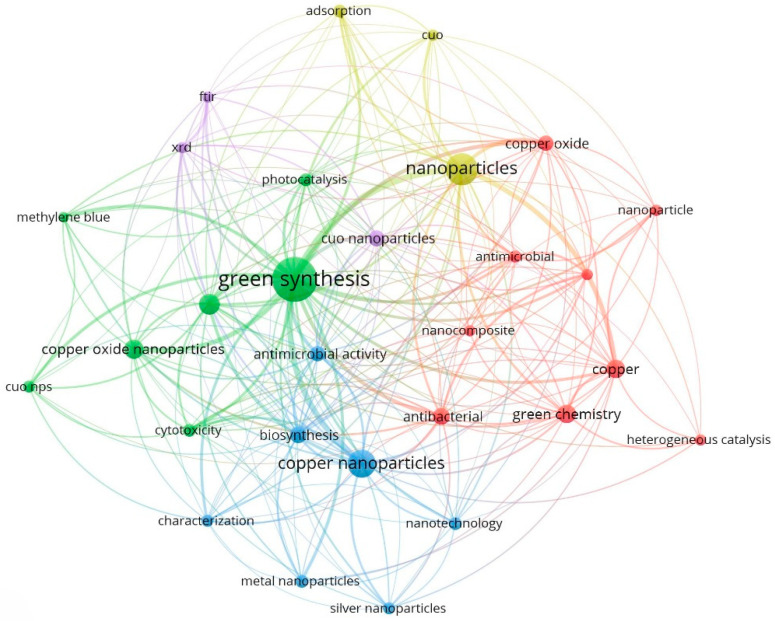
VOSviewer visualization of a term co-occurrence network based on the title and abstract fields, narrowing 30 keywords and 29 co-occurrences.

As shown in Figure 1, the bibliographic search for the diagnosis of AD resulted in 4098 articles. After removing the duplicates, unrelated topics, and other parameters, a total of 192 publications were selected for complete analysis. The studies examined were related to antioxidant capacity (20.31%), antitumoral (21.88%), antibacterial (27.08%), and catalytic effects (30.73%). The minimum number of occurrences of keywords was set at 30 keywords, and co-occurrences were generated 29 times (Figure 1). 

### 2.1. The Antioxidant Effect of CuNPs

**Figure 2:**
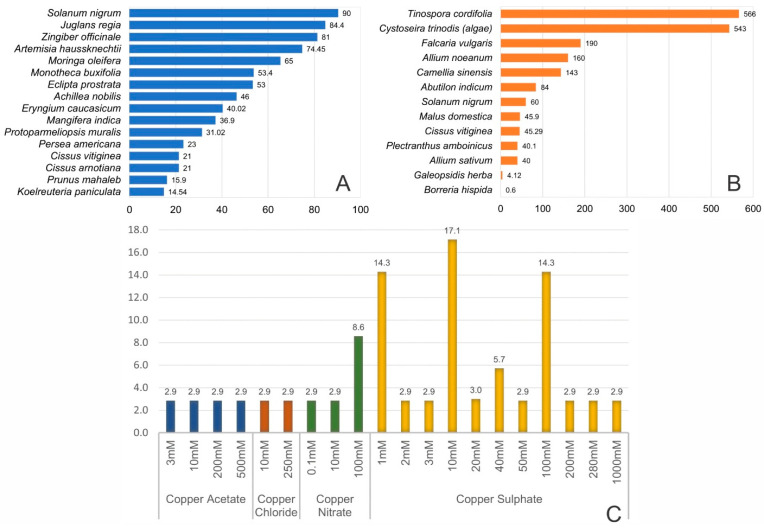
Antioxidant effect of CuNPs synthesized with biological extracts; (**A**) **a**ntioxidant activity (%), (**B**) IC50 (µg/mL), and (**C**) frequency of salt and concentrations of articles reviewed.

The antioxidant properties of CuNPs synthesized from plant extracts have been demonstrated. Figure 2 reveals that a determining factor was the salt concentration, which, despite being variable, ranged from 1 to 100 mM in most cases (Figure 2C). According to the research, the antioxidant capacity depends on the concentration due to the same behavior observed in metallic salts, such as copper acetate, copper chloride, and copper nitrate. However, copper sulfate exceeds these values [16].

Furthermore, the percentage of the antioxidant activity of 16 plants was screened by analyzing the antioxidant effects of CuNPs in each of the papers reviewed: this quantity is due to the complete available information. The antioxidant activity of *Koelreuteria paniculata* seed extract (14.54%) was lower than that of *Solanum nigrum* leaf extract (90%) (Figure 2A). Despite these values, the half maximal inhibitory concentration (IC_50_) of *Tinospora cordifolia* extracts was higher (566 µg/mL) than those of *Solanum nigrum* leaf extract*s* and *Borreria hispida* (60 µg/mL and 0.6 µg/mL, respectively) (Figure 2B).

Table 1 presents an overview of different metallic salts and concentrations, with various plants and the component extract used in CuNPs. As a result, the part of the plant used for extract preparation, such as leaves, fruits, stems, roots, flowers, and seeds, is significant because of the relationship between the phytochemical characteristics and the total antioxidant capacity necessary for the reduction to synthesize CuNPs. Because phenolic components are considered the main contributors to plant extracts’ total non-enzymatic antioxidant capacity, a decrease in phenolic compounds most likely resulted in reduced radical scavenging capacity [17]. In some cases, such as with tree bark, a direct relationship between antioxidant activity and total polyphenolic content was observed [18]. It is important to note that the phytochemicals present in the extract can be used as reducing and stabilizing agents in the overall synthesis [14].

In contrast, the antioxidant effect underlies the inhibition of chain reactions, such as the breakdown of peroxides, the binding of transition metal ion catalysts, radical scavenging activity, and the inhibition of continued hydrogen abstraction [14]. The antioxidants present in plants can act as stabilizing agents during synthesis, preventing nanoparticle aggregation or clustering. These plant components with antioxidant activity can reduce oxidative stress during synthesis. Oxidative stress can lead to the formation of ROS, which can be detrimental to the synthesis and stability of nanoparticles [14].

### 2.2. The Antitumoral Effect of CuNPs

#### 2.2.1. Anticancer Activity of CuNPs

In recent decades, radiotherapy, chemotherapy, and surgery have been the options with which to treat cancer. However, these standard methods have cost and usage restrictions. Thus, a natural, cheap, non-toxic, and side-effect-free treatment and prevention option is urgently needed. The Food and Drug Administration of the United States (FDA) recognizes metallic nanoparticles (i.e., iron, gold, zinc, titanium, and silver) as safe therapeutic compounds. Thus, CuNPs (374 USD/lb) have attracted considerable interest from researchers because they are cheaper than gold (1973 USD/t oz), silver (24.17 USD/t oz), and platinum (997 USD/t oz) [54]. Therefore, approaches involving CuNPs will be highly cost-effective [16].

CuNPs are promising cancer diagnosis and evaluation agents. Due to their unique properties, including their high surface-to-volume ratio, diffusion, efficiency of synthesis, and optical properties, they are effective against many cancer cell lines. These crucial factors are essential for extending the drug’s half-life and delivery application [55].

Depending on the source of CuNPs and the type of cell lines, CuNPs can act via various cytotoxic mechanisms, primarily ROS production, apoptosis, autophagy, and DNA damage. CuNPs interact actively with intracellular protein functional groups, nitrogen bases, and phosphate groups in DNA, causing cytotoxicity in tumor cell lines. Nanoparticles with anticancer properties are known for their potential ability to inhibit abnormally expressed signaling proteins, such as Akt and Ras, cytokine-based therapies, DNA- or protein-based vaccines against specific tumor markers, and tyrosine kinase inhibitors with a consistent antitumor effect [56].

In vitro research of various human cell lines, including neuronal cells, cardiac microvascular endothelial cells, kidney cells, liver cells, and lung epithelial cells, demonstrated that oxidative stress mediates the cytotoxicity of CuNPs. Thus, the excessive use and disposal of CuNPs increase their potential toxicity to the environment and human health. Therefore, the biocompatibility of synthesized CuNPs must be determined [57]. The high concentration of free radicals in normal cells causes numerous mutations in their DNA and RNA, accelerating the proliferation and growth of abnormal or cancerous cells. 

Some reports have suggested that CuNPs could induce apoptosis in cancerous cells via ROS generation by modulating the uptake of P53 and Bax/Bcl-2. Previous research indicates that the mechanism of action involves the destruction of ROS generated during cancer cell proliferation and transported as radicals or free radicals [58]. Regarding the ROS species (·OH, ·O_2_, and H_2_O_2_), they play a crucial role in the death of eukaryotic cells induced by biogenic CuNPs. The highly reactive ·OH is a significant oxidant that influences oxidative DNA damage, including single- and double-strand breaks, mutation discovery, and the production of oxidized nucleotides [59].

The anticancer efficacy of CuNPs seems to depend on their size, morphology, specific surface area, increase in oxygen vacancies, reactant molecule diffusion ability, and release of Cu^2+^. When particle size decreases, particle surface area increases dramatically. It increases the potential number of ROS groups on the particle surface, which could substantially impact adverse biological effects [60]. In addition, small NPs offer a larger surface area to produce ROS, such as hydrogen peroxide, superanion radicals, and hydroxyl radicals, in cancer cells [57]. The smaller size of CuNPs may result in widespread tissue distribution, deeper penetration within specific tissues, improved cellular uptake, and enhanced cytotoxicity against cancer cells [61]. Research shows particles smaller than 50 nm exhibit more activity in the different cancer cell lines [28]. NPs with dimensions less than 200 nm exhibit efficient extravasation into leaky tumor vasculature and accumulation in tumor tissues due to their increased permeability and retention effect [62]. In vivo studies on the biodistribution and toxicity of CuNPs have revealed that smaller particle sizes exhibit greater transvascular and interstitial transport. In tumors, 50 nm NPs have demonstrated significantly greater permeability than 125 nm NPs [63]. Therefore, an efficient drug carrier must be small enough to leave the bloodstream, enter the vessels, and reach the tumor site [64]. 

#### 2.2.2. Cytotoxic Effect of CuNPs on Cancer Cell Lines

The cytotoxic effects and biocompatibility of CuNPs depend on their concentration and synthesis routes. The synthesis of CuNPs has been reported mainly by physical methods, like ball milling, chemical methods, such as the sol-gel method, and biological methods by different plants or animal extracts. Among these routes, the biological routes, often called ‘green synthesis’, have proven to be one of the most biocompatible and ecofriendly methods for CuNP synthesis due to using eco-compatible reagents for the synthesis process [65]. Both the synthesis route and the inherent nature of CuNPs are important considerations for their effectiveness. The choice of synthesis route affects their specific characteristics, while their inherent nature determines their functional properties and behavior in specific applications.

Table 2 shows different plants that have been used as biological sources for the synthesis of CuNPs that have a cytotoxic effect on cancer cell lines.

CuNPs became of great interest due to their cytotoxicity for multiple types of cancer in a dose-dependent manner without affecting healthy cells, compared to chemically synthesized NPs, which cause cell death in both benign and cancerous cells.

(a) CuNPs against breast cancer

The effects of CuNPs derived from *Prunus nepalensis* on MCF7 were studied by analyzing the expression of oncogenes (Ras, Myc) and tumor suppressor genes (P14/P19, P53, P21, and Caspase 3). The results demonstrated that CuNPs increased the expression of the genes involved in apoptosis in a dose-dependent manner. Furthermore, CuNPs induced apoptosis in MCF-7 cells by downregulating oncogenes and upregulating tumor suppressor genes [56]. Additionally, XRD studies on CuNPs synthesized from *G. Sylvestre* leaf extract showed a smaller crystal size with a higher surface area, which increased their anticancer activity. The cytotoxicity results showed that green CuNPs were more effective against MCF-7 cells than chemically synthesized CuNPs [60].

The cytotoxicity of CuNPs derived from *Echinophora platyloba* was observed in Raji and MCF-7 cells. The interaction of CuNPs with circulating tumor DNA (ct-DNA) showed an unusual binding mode that combines the characteristics of groove binding and intercalation, suggesting that the cytotoxicity of CuNPs may result from their interaction with DNA [66].

Additionally, the treatment of MDA-MB-231 cells with CuNPs resulted in distinct morphological changes, such as shrinkage, detachment, membrane blebbing, and distortion [61].

On the other hand, it uses an endophytic bacterium. A low concentration of green synthesized CuNPs (2–28 nm) has antiproliferative effects on breast cancer (T47D) cell lines [67].

(b) CuNPs against cervical cancer

CuNPs were synthesized by using the aqueous leaf extracts of *Azadirachta indica*, *Hibiscus rosa-sinensis*, *Murraya koenigii*, *Moringa oleifera*, and *Tamarindus indica* and were tested against cervical cancer cells, revealing changes such as caspase activation, plasma membrane blebbing, binucleation, cytoplasmic vacuolation, cell shrinkage, nuclear fragmentation, chromatin condensation, chromosomal apoptotic bodies, and DNA fragmentation. The IC50 ranged between 20.32 to 26.73 µg/mL, with an average size of 12 nm [19]. Another study used black beans to produce NPs that caused cell death by activating apoptotic pathways initiated by intracellular ROS [68].

Similarly, CuNPs 40–45 nm in size were synthesized using *Houttuynia cordata* extract. The fluorescent staining analysis revealed the inhibition of cell proliferation and promotion of apoptotic cell death in HeLa cells targeting the PI3K/Akt signaling pathways at 5 and 7.5 µg/mL doses with an IC50 of 5 µg/mL [69].

(c) Copper/copper oxide NPs against lung cancer

The cytotoxic effects of CuNPs were examined in A549 cells, where they induced apoptotic pathways and caused low cell viability with fragmented nuclei and loss of membrane integrity. Aqueous Ficus religiosa leaf extract was used to synthesize copper NPs, showing that, at higher concentrations (500 g/mL), cell viability was reduced by up to 6%. The presence of bioactive molecules in *F. religiosa* leaf extract may explain the improved cytotoxicity; in conclusion, copper oxide nanoparticles activate the apoptosis pathway via the generation of reactive oxygen species, and mitochondrial depolarization causes cell death via nuclear fragmentation [70].

Similarly, as previously stated, copper oxide NPs synthesized by the aqueous leaf extracts of *A. indica*, *H. rosa-sinensis*, *M. koenigii*, *M. oleifera*, and *T. indica* showed a cytotoxic effect on lung cancer cells. The induction of apoptosis was accompanied by membrane blebbing, cell shrinkage, caspase activation in the cytoplasm, and nuclear fragmentation [19].

(d) Copper/copper oxide NPs against ovarian cancer

Green CuNPs synthesized from *Camellia sinensis* aqueous leaf extract showed high cell death and anti-human ovarian cancer properties against CAOV-3, SW-626, and SK-OV-3 cell lines. Even at high doses, the healthy cells were unharmed [53]. In another study, small CuNPs were obtained (6–15 nm) using *Cressa* leaf extract, inhibiting SKOV3 human ovarian carcinoma cell line growth with an IC 20 value of µg/mL [71]. 

*Olea europaea* leaf extracts had the highest inhibition yield (94%) at 50 g/L. CuNPs targeted AMJ-13 and SKOV-3 cell lines and induced cell shape changes, clumping, and cell communication inhibition [72].

(e) CuNPs against other cancer cells lines

CuNPs synthesized from plant extracts showed promising results against other cancer cells. For instance, pumpkin seed extract was used to synthesize CuNPs against colorectal cancer cells (HCT-116) [73], in which the results showed significant apoptotic induction after treating the cells with 25 g/mL CuNP. CuNPs made from *Allium noeanum* extract against three endometrial cancer lines (Ishikawa, HEC-1-A, HEC-1-B, and KLE) showed that the percentage of cell viability decreases when increasing CuNP concentration [28]. In the case of melanoma (B16F10)-synthesized CuNPs from *Quisqualis indica* floral extract, the results indicate that CuNPs induced cytotoxicity via apoptosis involving LDH release, ROS generation, and GSH depletion in a dose-dependent manner [74]. 

In contrast, when CuNPs were synthesized using *Vibrio* sp. to research cytotoxicity activity in esophageal cancer cells (KYSE30), the viability of KYSE30 cells was significantly reduced over time [IC50 = 37.52 mg/L (24 h), IC50 = 18.26 mg/L (48 h), and IC50 = 13.96 mg/L (72 h)] [75]. In another study using the fresh biomass of the cyanobacteria *Cylindrospermum stagnale*, CuNPs were found to significantly increase the concentration in a time-dependent manner. At 24 h, increasing the particle concentration from 25 to 100 μg/mL increased cell viability inhibition from 45 to 66% [76].

**Figure 3:**
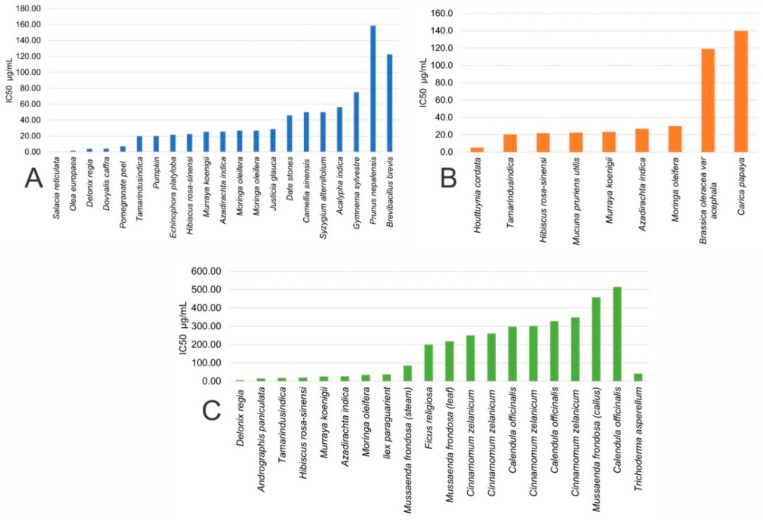
Antitumoral effect (IC50) of CuNPs synthesized with biological extracts in (**A**) breast, (**B**) cervical, and (**C**) lung cancer cell lines in vitro.

As shown in Figure 3, the IC50 antitumor effect on the major types of cancer cell lines is demonstrated using biological sources, mainly plant extracts.

Additionally, in human bone marrow mesenchymal stem cells, studies on the effect of CuNPs have demonstrated a certain suppression of proliferation development due to the activation of apoptotic pathways [77]. Nonetheless, numerous other studies report a positive effect when applied to biomaterials, primarily due to osteogenic properties for the regeneration of bone tissue and cartilage, where an increase in porosity, mechanical strength, and cross-linking is observed in scaffolds. These results and those of a number of other authors are contradictory, indicating that the topic must be explored and studied further, as it appears that determining the appropriate forms and concentration of copper is essential for their safe and effective application [78,79,80].

**Table 2:** The antitumoral effect of biogenically synthetized CuNPs, salt, concentration, and biological source.

Plant	Part	Metallic Concentration	Cell Line	Size (nm)	Assay	IC50 (µg·mL^−1^)	Reference
Breast cancer							
Copper sulfate							
*Syzygium alternifolium*	Stem bark	5 mM	(MDA-MB-231)	5–13	MTT	50	[81]
*Olea europaea*	Leaves	2 mM	(AMJ-13)	20–50	MTT, AE-EB	1.47	[72]
*Justicia glauca*	Leaves	0.1 M	(MCF-7)	19.72	MTT	28.72	[82]
*Dovyalis caffra*	Leaves	1 mM	(MCF-7)	30–50	MTT	4.04	[58]
*Punica granatum*	Peel	1.0 M	(MCF7)	6	MTT	7.1	[83]
*Phoenix dactylifera*	Pits	1.0 M	(MCF7)	20	MTT	45.7	[83]
*Prunus nepalensis*	Fruit	1 mM	(MCF-7)	35–50	MTT	158.5	[56]
*Cystoseira myrica*	Algae	1 mM	(MCF-7)	21	MTT	-	[84]
*Delonix regia*	Leaves	5 mM	(MCF-7)	69–108	MTT	3.77	[85]
*Acalypha indica*	Leaves	-	(MCF-7)	26–30	MTT	56.16	[86]
Copper acetate							
*Cucurbita* spp.	Seed	3 mM	(MDA-MB-231)	20	MTT, AO-EB, ROS, MMP	20	[61]
*Hibiscus rosa-sinensi*	Leaves	0.2 M	(MCF-7)	12	MTT, Hoechst 33258	22.45	[19]
*Murraya koenigii*	Leaves	0.2 M	(MCF-7)	12	MTT, Hoechst 33258	25.32	[19]
*Moringa oleifera*	Leaves	0.2 M	(MCF-7)	12	MTT, Hoechst 33258	26.71	[19]
*Tamarindusindica*	Leaves	0.2 M	(MCF-7)	12	MTT, Hoechst 33258	19.77	[19]
*Brevibacillus brevis*	Biomass	100 ul	(T47D)	2–28	MTT	122.3	[67]
*Camellia sinensis*	Leaves		(MCF-7)	22	MTT	50	[87]
*Azadirachta indica*	Leaves	0.2 M	(MCF-7)	12	MTT, Hoechst 33258	25.55	[19]
Copper nitrate							
*Salacia reticulata*	Leaves	1 mM	(MCF-7)	42	MTT	0.42	[88]
*Echinophora platyloba*	-	0.1 M	(MCF-7)	10	MTT, AO/BE	21.44	[66]
Cervical cancer							
Copper sulfate							
*Houttuynia cordata*	Plant	3 mM	(HeLa)	40–45	MTT, AO/EtBr	5	[69]
*Black beans*	Beans	10 mM	(HeLa)	26	SRB, ROS, MMD, Clonogenic survival	-	[68]
*Brassica oleracea var acephala*	Leaves	1 mM	(HeLa)	60–100	MTT	119.0	[89]
*Carica papaya*	Leaves	5 mM	(HeLa)	77	MTT	139.	[90]
*Mucuna pruriens utilis*	Seed	2.5 gr	(HeLa)	28	MTT	22.48	[91]
Copper acetate							
*Azadirachta indica*	Leaves	0.2 M	(HeLa)	12	MTT, Hoechst 33258	26.73	[19]
*Hibiscus rosa-sinensi*	Leaves	0.2 M	(HeLa)	12	MTT, Hoechst 33258	21.63	[19]
*Murraya koenigii*	Leaves	0.2 M	(HeLa)	12	MTT, Hoechst 33258	23.22	[19]
*Moringa oleifera*	Leaves	0.2 M	(HeLa)	12	MTT, Hoechst 33258	30.08	[19]
*Tamarindusindica*	Leaves	0.2 M	(HeLa)	12	MTT, Hoechst 33258	20.32	[19]
Epithelioma							
Copper acetate							
*Moringa oleifera*	Leaves	0.2 M	(Hep-2)	12	MTT, Hoechst 33258	29.58	[19]
*Tamarindusindica*	Leaves	0.2 M	(Hep-2)	12	MTT, Hoechst 33258	21.66	[19]
*Azadirachta indica*	Leaves	0.2 M	(Hep-2)	12	MTT, Hoechst 33258	28.59	[19]
*Hibiscus rosa-sinensi*	Leaves	0.2 M	(Hep-2)	12	MTT, Hoechst 33258	22.59	[19]
*Murraya koenigii*	Leaves	0.2 M	(Hep-2)	12	MTT, Hoechst 33258	25.59	[19]
Hepatocellular carcinoma							
Copper acetate							
*Eclipta prostrata*	Leaves	3 mM	(HepG2)	23–57	MTT	-	[20]
*Curcuma longa*	Root	6 gr	(HepG2)	27	MTT	64.10	[92]
*Azadirachta indica*	Leaves	2 gr	(HepG2)	15–16	MTT	-	[93]
*Cylindrospermum stagnale (cyanobacteria)*	Biomass	1 mM	(HepG2)	12	MTT	-	[76]
Copper sulfate							
*Cystoseira myrica*	Brown alga	1 mM	(HepG2)	21	MTT	-	[84]
*Momordica cochinchinensis*	Leaves	0.01 M	(HepG2)	56	MTT, ROS	75	[94]
Lung carcinoma							
Copper acetate							
*Andrographis paniculata*	Leaves	0.5 M	(A549)	23	MTT	14.76	[59]
*Tamarindusindica*	Leaves	0.2 M	(A549)	12	MTT, Hoechst 33258	18.11	[19]
*Murraya koenigii*	Leaves	0.2 M	(A549)	12	MTT, Hoechst 33258	25.05	[19]
*Moringa oleifera*	Leaves	0.2 M	(A549)	12	MTT, Hoechst 33258	34.37	[19]
*Azadirachta indica*	Leaves	0.2 M	(A549)	12	MTT, Hoechst 33258	26.03	[19]
*Hibiscus rosa-sinensi*	Leaves	0.2 M	(A549)	12	MTT, Hoechst 33258	20.15	[19]
Copper sulfate							
*Ficus religiosa*	Leaf	5 mM	(A549)	577	MTT, AO/BE	200	[70]
*Delonix regia*	Leaves	5 mM	(A549)	69–108	MTT	4.70	[85]
*llex paraguariensist*	Leaves	1 mM	(A549)	26–40	MTT	36.89	[95]
Copper nitrate							
*Trichoderma asperellum*	Cell-freeextract	5 mM	(A549)	110	WST-1	40.625	[96]
*Cinnamomum zelanicum*	Leaves	0.3 M	(NCI-H2126II, NCI-H1437.III, NCI-H1573.IV, NCI-H661)	9–69	MTT	250, 348, 301, and 261	[97]
*Mussaenda frondosa*	Leaf, stem and callus	-	(A549)	2–10	MTT, Dual AO/EB	85.66–458.35	[98]
*Calendula officinalis*	Leaves	0.3 M	(LC-2/ad, PC-14, HLC-1)	19–39	MTT	328, 297 and 514	[55]
Colorectal cancer							
*Carica papaya*	Leaves	Copper sulfate 5 mM	(HT-29)	77	MTT	93.	[90]
*Ormocarpum cochinchinense*	Leaves	Copper chloride 0.003 M	(HCT-116)	1–2	MTT	40	[99]
*Cucurbita maxima*	Seed	Copper acetate 3 mM	(HCT-116)	20	MTT, AO/BE	25	[73]
Ovarian cancer							
*Olea europaea*	Leaves	Copper sulfate 2 mM	(SKOV-3)	20–50	MTT, AO-EB	2.27	[72]
*Camellia sinensis*	Leaves	Copper chloride 1.7 gr	(CAOV-3, SW-626, and SK-OV-3)	10–20	MTT	263, 208 and 315	[53]
*Cressa spp.*	Leaves	Copper sulfate	(SKOV3)	6–15	MTT	20	[71]
Other types of cancer							
*Brassica oleracea*	-	Copper sulfate 5 mM	Prostate cancer (PC-3)	4	-	-	[100]
*Echinophora platyloba*	-	Copper nitrate 0.1 M	Raji Burkitt’s Lymphoma	10	MTT, AO/BE	10.79	[66]
*Allium noeanum*	Leaves	Copper nitrate 1 mM	Endometrial Cancer (Ishikawa, HEC-1-A, HEC-1-B,and KLE)	10–12	MTT	357, 356, 331 and 411	[28]
*Vibrio spp.*	Bacteria	Copper nitrate	Esophageal Cancer (KYSE30)	8	MTT	18.26	[75]
*Rhus punjabensis*	-	-	Leukemia (HL-60)	31–36	SRB	1.82	[101]
*Citrus aurantifolia*	Enzyme	Copper sulfate 1 mM	Melanoma (SK MEL 28)	4	MTT	56.6	[102]
*Quisqualis indica*	Floral	Copper acetate 5.0 mM	Melanoma (B16F10)	39	MTT, LDH	102	[74]
*Arbustus unedo*	Leaves	Copper sulfate 0.01 mM	Nasopharynx cancer (KB)	30	MTT	-	[103]
*Aerva javanica*	Leaves	Copper chloride 4 mM	Neuroblastoma (Neuro2A)	15–23	MTT	-	[57]
*Rhus punjabensis*	-	-	Prostate adenocarcinoma (PC-3)	31–36	SRB	19.25	[101]

MTT: 3-[4,5-dimethylthiazol-2-yl]-2,5 diphenyl tetrazolium bromide assay, LDH: Cytosolic lactate dehydrogenase assay, SRB: Sulforhodamine B assay, AO/BE: Acridine orange/ethidium bromide staining, WST-1: Water-soluble tetrazolium salt assay, ROS: total reactive oxygen species (ROS) assay, MMD: mitochondrial membrane depolarization assay, MMP: mitochondrial membrane potential, AO/EtBr: acridine orange/ethidium bromide assay.

### 2.3. The Antibacterial Effect of CuNPs

The antimicrobial effect of nanoparticles on bacteria is due to the effect caused at the cell wall level, primarily composed of the polymers of peptidoglycans, sugars, and amino acids, which, due to their porosity, facilitate the passage of nanoparticles [104]. This effect is also highly dependent on the type of bacteria: Gram-negative cell walls comprise a single layer of peptidoglycan, whereas Gram-positive cell walls comprise multiple layers [26]. Gram-negative bacteria are less resistant due to greater negative surface charge [105].

Gram-positive micro-organisms have walls composed of amines and carboxyl groups, which, when combined with aryls in the presence of copper, result in an amination reaction, and increased membrane permeability [106,107].

The inhibitory or bactericidal effect of CuNPs results precisely from the inhibition of cell membrane enzymes caused by the attraction between NPs and the membrane, thereby promoting the oxidation of NPs that are electrostatically attracted to membrane-based plasma reductases [108]. These ions enter through the lipid layers and move towards the cytosol, causing the production of oxygen species as O_2_, which leads to the formation of H_2_O_2_, which is responsible for the oxidation of proteins and lipids [109,110].

According to the bioinformatic results of Ul-Hamid et al., 2022 [111], the CUNP binds to the Ile14, Thr12, GLN95, PHE92, Tyr98, Thr46, and Thr121 residues of *S. aureus* dihydrofolate redrawn tRNA synthetase and dihydropteroate synthetase, consequently inhibiting the activity of these enzymes.

Particularly when nanoparticles are small and spherical, their size and shape could have potential as inhibitors [112]. Nanoparticles enhance antimicrobial activity and membrane performance by increasing the surface area [113]. After interacting with compounds, such as sulfur and phosphorus, the nanoparticles appear to have introduced reactive hydroxyl radicals capable of causing irreversible damage, oxidizing the proteins, and causing damage at the RNA and DNA levels, thereby altering and destroying the helical structure [114].

The phytochemical composition of the source (such as phenolic compounds and other antioxidants) that allows for the synthesis and stability of the nanoparticles will significantly impact these characteristics and effects [115]. CuNPs combined with plant extracts can further enhance the antimicrobial effect due to terpenoids (present in essential oils), phenolic compounds, tannins, flavonoids, and alkaloids that can cause ion transport disruptions and alter the activity of ion transport [116,117].

The destruction of proton efflux bombs results in the release of toxic metal ions, which affects the permeability of pathogen membranes and the respiratory system [118].

According to research by Selvan et al., 2018 [118], CuNPs have an anti-larvicidal effect on the *Anopheles subpartus* due to their accumulation in the alimentary and respiratory channels, which causes a rupture in the layers of tissues.

Alternatively, when compared to bacteria, fungi can be less sensitive to CuNPs due to the nature of their cell walls. Fungi have cell walls composed of polysaccharides, such as chitin (N-acetylglucosamine) and lipids, which provide stiffness and resistance to nanoparticles [119].

Table 3 includes columns for salt concentration, shape, and biological source, allowing for a comprehensive analysis of the antibacterial effect in different micro-organisms.

### 2.4. The Catalytic Effect of CuNPs

The catalytic effect depends on chemical production, elimination, and industrial catalytic process efficiency, selectivity, and yield. Selectivity reduces waste and impurities, making products safer and greener [170]. Copper oxide nanoparticles are reactive metal oxide semiconductors. Their high surface area facilitates catalytic, antimicrobial, and antifouling effects [171]. They are relevant in two areas: a reduction in components, characterized by the reduction or elimination of dyes and colorants, and the synthesis and catalysis of elements. Table 4 summarizes the screened data.

When a dye solution containing nanoparticles is exposed to sunlight, a free electron and a hole are generated from the nanoparticles; the electron interacts with oxygen to form superoxide free radicals, whereas the hole interacts with water to form hydroxyl ions. The dye is decolored by the formed superoxide free radicals and hydroxyl ions [172].

Each reaction or product has its pathway in component synthesis and catalysis. Quantum effects and a large surface-to-volume ratio give metal nanoparticles fascinating UV–visible, catalytic, and antibacterial properties [173]. The catalytic activity might be affected by nanoparticle size [42], shape, and exposed crystal planes [32]. CuNPs with a high surface area are popular due to their stability, cost, toxicity, manufacturability, and potential as catalysts [174].

#### 2.4.1. Reducing and Capping Agents in the Synthesis of CuNPs

The reducing agent is an important component of nanoparticle synthesis. All research is included due to the ecofriendly approach, where the preference of agents derived from micro-organisms or plant species is critical. Thus, this review covers some of the most important nanoparticle-synthesizing plants. Most of the papers in this section use agricultural waste like calli [98], rhizomes [175,176], fruit hulls [177], aerial parts [178], beans [179], leaves, peels, flowers, juice, and peels [180]. There are also some byproducts, such as gum [181], and some species from another kingdom, such as the algae *Cystoseira trinodis* [42] and the bacteria *Escherichia* sp. *SINT7* [182]. We can also highlight the application of plants like *Plukenetia volubilis* [183] and *Moringa oleifera* [184], which are widely cultivated in Peru and can be used for CuNP synthesis.

#### 2.4.2. Factors That Affect the Synthesis and Catalysis of CuNPs

(a) Particle size: Most of the papers reviewed have synthesized nanoparticles with a maximum size of 100 nm, noting that decreasing particle size will increase catalytic activity [185] and that the solvent concentration affects the particles’ size and shape [186]. Furthermore, it has been discovered that a larger extract volume is required to produce nanoparticles with a narrower size range [155];
(b) Temperature: The temperature of the reaction is an essential factor to consider when synthesizing nanoparticles. Some of the papers reviewed emphasized the importance of a high temperature for synthesis;
(c) pH: pH is essential in nanoparticle synthesis, ranging from 9 [155,187] to 10 [188] and to 12 [172,189].

**Table 4:** The catalytic application of biogenically synthetized CuNPs, salt, concentration, and biological source.

Scientific Name	Source	Concentration	Size (nm)	Catalytic Application/Results		Reference
				Compound Synthesis, Degradation/Yield (%)	Compound Removal/Yield (%)	
Copper nitrate						
*Carica papaya*	Fruit peels	0.004 M	28.0	Palm oil effluents degradation: 66%.	ND	[190]
*Sacha inchi*	Leaves	0.01 M	8–32	ND	MB degradation: 78.90%.	[155]
*Camelia sinensis* and *Prunus africana*	Leaves	0.0005 M	6–8	ND	MB degradation: 83–85%	[191]
*Aglaia elaeagnoidea*	Flowers	0.001 M	20–45	Reduction of 4-NP to 4-AP: ~99%.	MB degradation: yield ~99%. CR degradation: yield ~99%.	[171]
*Achyranthes aspera* and *Crotalaria verrucosa*	Leaves	0.003 M	10–20	ND	RhB degradation: 91–96%.	[192]
*Cordia sebestena*	Flowers	0.1 M	20–35	Synthesis of pyrimidinones: 98%. DHPM derivatives synthesis: 98% ^(case VIII)^.	BTB dye degradation: ~99%	[131]
*Solanum nigrum*	Leaves	0.1 M	25	ND	MB degradation: 97%.	[26]
*Moringa oleifera*	Leaves	0.1 M	28	ND	Pararosaniline dye degradation: 96.4%.	[184]
*Passiflora edulis*	Leaves	0.1 M	60–65	ND	MB degradation: 93%.	[131]
*Citrofortunella microcarpa*	Leaves	1 M	54–68	ND	Rhodamine degradation: 98.31%.	[193]
*P. emblica*	Leaves	26.7 M	80	ND	As (V) removal: 98.9%	[194]
*Musaenda frondosa*	Callus	-	2.2	ND	MB degradation: 88–97%	[98]
*Rauvolfia serpentina*	Leaves	-	10–20	ND	TB dye degradation: ~99%	[134]
Copper acetate						
*Syzygium jambos* (L.) *Alston*	Leaves	0.001 M	7.62	Ipso-Hydroxylation of aryl boronic acids:100% ^(case IV,XIV)^.	ND	[195]
*I. tinctoria*	Leaves	0.001 M	10–30	Synthesis of 2-(benzo[d]thiazol-2-ylthio) benzoic acid: 65–80% ^(case II)^.	ND	[196]
*Thymbra spicata*	Leaves	0.001 M	10–20	Reaction of aniline with 4-chlorobromobenzene: 96%	ND	[197]
*Stachys Lavandulifolia*	Flowers	0.001 M	20–35	Thyoldibenzene synthesis: 96%. Benzenethiol synthesis: 98%.	ND	[198]
*Salvia hispanica*	Leaves	0.1 M	30	Cycloaddition of alkyl halides: 93% for CuO NPs, 99% for Cu_2_O NPs	MB degradation: ~99%	[43]
*Ocimum tenuiflorum*	Leaves	0.1 M	6–18	ND	MO degradation: 96.4%.	[87]
*Mimosa pudica*	Leaves	0.1 M	8	Reduction of p-nitrophenol: ~99%	ND	[199]
*Quercus infectoria*	Fruit	0.2 M	26	ND	BV 3 dye degradation: 86%	[177]
*Citrus limon*	Juice	0.3 M	5–20	ND	Cr (VI) adsorption: 98.3 ± 0.6%	[200]
*Lantana camara*	Flowers	0.4 M	13–28	Aza Michael reaction: 80%. Enamine production: 90% ^(case VI)^.	ND	[189]
*Andrographis paniculata*	-	0.5 M	23	ND	MB degradation: 98%.	[59]
*Psidium guajava*	Leaves	2 M	2–6	ND	NB degradation: 97%. RY160 degradation: 80%.	[201]
Copper chloride						
*Gundelia tournefortii*	Leaves	0.003 M	ND	Hydration of cyanamides: 89%. Reduction of 4-nitrophenol: ~99%	ND	[202]
*Tamarix galica*	Leaves	0.003 M	Various sizes	N-arylation of triazoles: 92% ^(case XII)^	ND	[174]
*Anthemis nobilis*	Flowers	0.003 M	38.6	A^3^ coupling reaction with piperidine: 89%	ND	[203]
*Thymus vulgaris* L.	Leaves	0.003 M	30	N-arylation of indoles: 97% ^(case V)^	ND	[204]
*Jatropha curcas*	Leaves	0.003 M	10	ND	MB catalysis: ~99%.	[205]
*Ageratum houstonianum*	Leaves	0.003 M	200	ND	CR degradation: ~99%	[206]
*Euphorbia esula* L.	Leaves	0.005 M	20–110	4 nitrophenol reduction: ~99%.	ND	[207]
*Ginkgo biloba* L.	Leaves	0.005 M	15–20	Reaction of benzyl azide with phenyl acetylene: 98% ^(case X)^.	ND	[208]
*Otostegia persica*	Leaves	0.005 M	ND	Synthesis of 1,2,3-triazoles: 93%	ND	[209]
*Plantago asiatica*	Leaves	0.005 M	7–35	Aldehyde cyanation: 93% ^(case IX)^.	ND	[210]
*Brassica oleraceae*, *Pisum sativum* and *Solanum tuberosum*	Peels	0.01 M	22–31	ND	MB degradation: 79–96%	[211]
*Coffea Arabica*	Beans	0.1 M	5–8	ND	AB 10B reduction: ~99%, MB and XO reduction: ~99%,	[179]
*Camellia sinensis*	Leaves	0.2 M	10–20	pyrano[2,3-d] pyrimidines synthesis: 90–98% ^(case XIII)^.	ND	[53]
*Punica granatum*	Seeds	1 M	40–80	ND	MB degradation: 87.1%	[212]
*T. Cordifolia*	Leaves		90	ND	Direct green, Eosin and Safranine and reactive dye: 90%	[213]
Copper sulfate						
*Convolvulus percicus*	Leaves	0.001 M	15–30	Arylation for C−N and C−O coupling reactions: 92%.	ND	[157]
*Odina wodier*	Gum	0.001 M	60–100	ND	Acid blue degradation: 96%.	[185]
*Alchornea laxiflora*	Leaves	0.001 M	3.2	Oxidative Desulphurization: 63.92% ^(case XI)^.	ND	[214]
*Cystoseira trinodis (algae)*	Biomass	0.001 M	7–10	ND	MB degradation: 98%.	[42]
*Euphorbia maculata*	Leaves	0.001 M	18.02	ND	MB degradation: 96%, CR degradation: 85%, RhB degradation: 89%	[178]
*Rosmarinus officinalis*	Leaves	0.001 M	ND	ND	MB degradation: 97.4%.	[215]
*Myrtus communis*	Leaves	0.001 M	55	4-nitrophenol (4-NP) reduction: ~99%.	ND	[188]
*Duranta erecta*	Fruits	0.005 M	70	ND	MO reduction: 96%, CR reduction: 90.35%	[216]
*C. epigaeus*	Rhizomes	0.005 M	65–80	ND	MB degradation: 90%.	[98]
*Manilkara zapota*	Leaves	0.005 M	18.79	ND	MV, MG, CBB degradation: 92.2%, 94.9%, 78.8%.	[172]
*A. muricata*	Leaves	0.005 M	30–40	ND	RR120 and MO degradation: 90%, 95%	[217]
*Escherichia* sp. *SINT7*	Biomass	0.005 M	30	ND	CR, MG, DB-1, RB-5 degradation: 97.0%, 90.5%, 88.4% and 83.6%	[182]
*Bergenia ciliata*	Rhizomes	0.005 M	50	ND	MB and MR degradation: 92%, 85%.	[176]
*Coccinia grandis*	Fruits	0.01 M	40–50	Reducing para-nitrophenol (PNP) to para-amino phenol (PAP): ~97%.	ND	[218]
*Aloe vera*	Leaves	0.01 M	24–61	ND	MB degradation: ~99%.	[186]
*Coccinia grandis*	Flowers	0.01 M	40–50	Reduction of 4-nitroaniline into amino compound: 97.9%.	ND	[219]
*Pterolobium hexapetalum*	Leaves	0.01 M	10–50	ND	RB 5 degradation: 98%.	[156]
*Triticum aestivum*	Seeds	0.01 M	22	Nitrophenol reduction: ~99%.	ND	[220]
*Diospyros montana*	Leaves	0.01 M	5.9–21.8	ND	MB degradation. ~99%.	[221]
*Cardiospermum halicacabum*	Leaves	0.01 M	14.9	ND	MB degradation: 93.6%.	[222]
*Mentha piperita*, *Citrus sinensis*	Leaves	0.01 M	150	ND	% Cd (II), Ni (II) and Pb (II) removal: 18%, 52.5%, 84%	[223]
*Impatiens balsamina*	Leaves	0.02 M	5–10	ND	MB degradation: ~85%, CR degradation: ~80%	[224]
*Z. spina-christi*	Fruit	0.02 M	9	ND	CV removal: 93.7%	[225]
*Citrus grandis*	Peel	0.04 M	22–27	ND	MR reduction: 96%.	[187]
*Elsholtzia blanda*	Leaves	0.05 M	32–49	ND	Degradation of CR dye: 74–94%	[226]
*Solanum lycopersicum*	Leaves	0.1 M	20–40	ND	Crystal 271 violet degradation: ~97%.	[146]
*Salvia hispanica*	Leaves	0.1 M	35	Cycloaddition of benzyl chloride: 99%	MB degradation: ~99%	[43]
*Centella asiatica*	Leaves	0.1 M	20–30	ND	MR and MO reduction: 98%, PR reduction:99.62%	[227]
*Aegle marmelos*	Peel	0.1 M	20	ND	MB degradation: ~99%.	[180]
*Clitoria Ternatea*	Flowers	0.1 M	18	ND	Cristal violet (CV), direct red DR degradation: 88.3%, 65%	[228]
*Citrus aurantifolia*	Leaves	0.3 M	55	ND	RhB dye degradation: 91%	[155]
*Musa balbisiana*	Peel	1 M	50–90	4-nitrophenol to 4-aminophenol conversion: 96% ^(case I)^.	ND	[229]
*Euphorbia maculata*	Leaves	1 M	18	C-S cross-coupling reaction: 89% ^(case VII)^	ND	[230]
*Aloe vera*	Leaves	-	80–120	ND	CR reduction: 70–75%	[231]

MB: methylene blue, CR: congo red, MB: methylene blue, MV: methyl violet, MG: malachite green, CBB: coomassie brilliant blue, NB: Nile blue, RY160: reactive yellow 160, MO: methyl orange, CV: crystal violet, DR: direct red, acid Blue 120, BTB: bromothymol blue, crystal 271 Violet, RB5: reactive black 5 dye, RhB: rhodamine B dye, NO: naphthol orange, MR: methyl red, RR: reactive red 120, DR: direct green, EY: eosin yellowish, TB: trypan blue.

In the process of the degradation and reduction of both dyes and organic compounds using CuNPs, nanoparticle and sample’ concentration, pH, time, and light are the parameters that must be considered in these processes. The best yields can be obtained using a higher nanoparticle concentration, a lower sample concentration [200], and a smaller nanoparticle size [185]. Alkaline pH improves the degradation or reduction process, and time in many studies is directly proportional to the light factor and the use of reducing agents.

**Figure 4:**
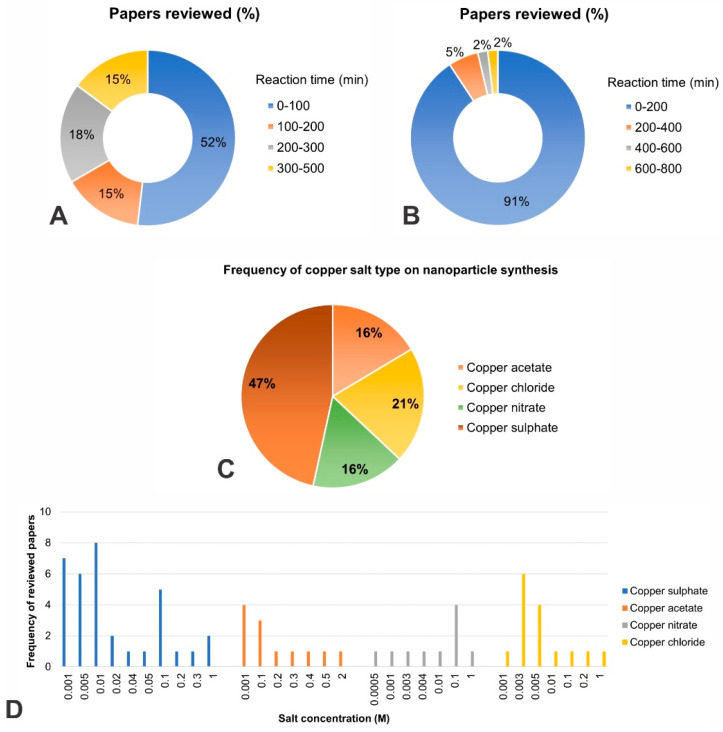
(**A**) Frequency (%) of papers reviewed of reaction time (min) on synthesis/catalysis/degradation/reduction approach; (**B**) Frequency (%) of reaction time (min) on dye removal approach; (**C**) Salt type frequency on nanoparticles synthesis (%), and (**D**) Salt concentration (M) frequency on nanoparticles synthesis.

(d) Reaction time: The reaction time was screened in all synthesis and dye removal papers. Regarding synthesis, most reviewed articles carried out a reaction time of 1 to 100 min. Reaction times of up to 200 to 500 min were also observed (Figure 4A). Regarding dye removal applications, most articles reported a time range of 1 to 200 min, and reaction times of up to 200 to 800 min were also observed. In both applications, most studies were successful in under 120 min (Figure 4B);
(e) Salt concentration frequency: The frequency of salt concentration was also screened through all the papers reviewed for catalytic application. The four main metallic salts synthesizing CuNPs were copper sulfate, copper acetate, copper nitrate, and copper chloride (Figure 4C). Copper sulfate was the most used salt, followed by copper chloride, copper nitrate, and copper acetate. The most frequently used concentration for copper sulfate was 0.01 M, copper acetate was 0.001 M, copper chloride was 0.003 M, and copper nitrate was 0.1 M. The concentration range for the most used salts, copper sulfate, and copper chloride ranged from 1 mM to 10 mM (Figure 4D).

#### 2.4.3. Mechanism of Compound Reduction Using CuNPs as a Catalyst

The reduction process consists of the following steps. The initial step involved the adsorption of reactants to the surface of nanoparticles. The adsorption of BH4- onto synthesized CuNPs transfers surface hydrogen to the nanoparticles. The second step is desorption, which produces a product on the nanoparticles’ surface. Immediately after the final product (amino compounds) desorption, the metal surface is made available for the catalytic cycle [219]. The pH of the solution primarily determines the removal of heavy metals from contaminated water [223]. CuNPs are an excellent alternative to adsorption for removing Cr (VI) toxicity from water [200]. Organic compounds require a reducing agent, such as NaBH4 or CuNPs, for a faster reduction [199]. One study found that using NaBH4 as a reducing agent under alkaline conditions reduced 97% of para-nitrophenol in 14 min [218].

Arsenic (V) was reduced by 98% in 50 min at pH 8 [194]; it was concluded that if no reducing agents were used, the treatment time would be longer. Lead is another heavy metal with a significant reduction percentage, with a value of 89% at pH 6. Mahmoud’s study discovered relatively low levels of cadmium and nickel, which could be attributed to their use of neutral pH rather than alkaline conditions. CuNPs are an excellent nanoabsorbent for purifying heavy metal-contaminated water, and their regeneration and reuse should be researched further [223].

Case I. Reduction of aromatic compounds: There are four steps in this reaction: hydrogen absorption, aromatic nitro compound absorption to metal surfaces, electron transfer from BH4- to aromatic nitro compounds, and aromatic amino compound desorption. Both azobenzene intermediates and hydroxyl amine reduction pathways most likely accomplish nitroarene reduction. The NPs (on their active surface) are reduced by the hydrogen liberated during sodium borohydride decomposition [229].

Case II. Production of benzoic acids: In the presence of NPs, the reaction occurs between 2-mercaptobenzothiazole and 2-iodobenzoic acid. Later, under optimized conditions, 3-iodo-5-nitro-1-tosyl-1H-indole (1 equiv.) and 2-fluoro-4-iodopyridine (1 equiv.) were reacted to give optimal (65–68%) yields for the coupling products 3a-c. 2-(benzo[d]thiazol-2-ylthio) benzoic acid was the product [196].

Case III. Synthesis of tetrazole derivatives: One specific example denotes preparing 5-(4-Nitrophenyl)-1H-tetrazole. The disappearance of reactants indicates a cycloaddition reaction, which is treated with muriatic acid and extracted with ethyl acetate. Finally, the light-yellow 5-(4-Nitrophenyl)-1H-tetrazole was obtained [232].

Case IV. Reduction of 4-nitrophenol: Another example of the catalytic properties of nanoparticles is the reduction of 4-NP to 4-aminophenol (4-AP). The result demonstrates that the natrolite zeolite/Cu NPs are required to reduce 4-NP to 4-AP [233].

Case IV. Homocoupling reactions: The base-free homocoupling reaction was also studied for various boronic acids. The reaction conditions are compatible with aryl functional groups such as aldehyde, ether, methyl, and nitro. Surprisingly, the bromo and chloro groups do not change. Other boronic acid surrogates, such as phenylboronic acid neopentylglycol ester, were also found to be suitable homocoupling reaction substrates [234].

Case V. N-arylation of compounds: It should be noted that the reaction of indole with iodobenzene was considered as a model reaction to determine the best reaction conditions for the catalytic N-arylation process [170].

Case VI. Aza-Michael reaction: The Aza-Michael reaction is ideal for creating the carbon–nitrogen (C–N) bond and other types of molecular bonds [189].

Case VII. Heterocoupling reactions: In organic synthesis, the Ullmann C−C homocoupling, C−N, and C−O hetero coupling reactions are required to prepare the bi-aryl, di-aryl amine, and di-aryl ether structures [235,236]. It should be noted that the C-S coupling reaction is also being investigated for thioether synthesis in the absence of a ligand [230].

Case VIII. Biginelli reaction: The catalytic efficacy of Cu/CuO/Cu_2_O nanoparticles in the Biginelli reaction to synthesize the biologically active compound 3,4-dihydropyrimidinone (DHPM). DHPM was prepared by loading various catalysts for the condensation of benzaldehyde with urea and ethylacetoacetate [131].

Case IX. Cyanation of aldehydes: This reaction occurs in the presence of non-toxic K4Fe(CN)6 as the cyanide source. The results confirmed the high stability and absence of impurities in reducing Cu II ions to CuO [210].

Case X. Huisgen [3 + 2] cycloaddition: To optimize the reaction conditions, the Huisgen [3+2] cycloaddition of azides and alkynes under ligand-free conditions with benzyl azide and phenyl acetylene [208].

Case XI. Oxidation of oils: As an oxidant, H_2_O_2_ can convert model oils (DBT in n-heptane) into the corresponding sulfone or sulfoxide. Peracetic acid and a CuNP oxide intermediate, hydroox-ocuprate, are formed by the nucleophilic attack of hydrogen peroxide on acetic acid and CuNPs, respectively [214].

Case XII. Synthesis of 1,2,3-triazoles: In synthesizing 1,2,3-triazoles, the reaction of benzyl chloride, sodium azide, and phenyl acetylene is critical [237].

Case XIII. Synthesis of pyrimidines: Another application of NPs as catalysts is the synthesis of pyrano[2,3-d] pyrimidines. Aromatic aldehydes, methylene compounds, barbituric acid, and Cu_2_O NPs were combined with solvents [53].

Case XIV. Ipso-hydroxylation of arylboronic acids: A typical reaction involves the ipso-hydroxylation of aryl and hetero-aryl boronic acids by phenyl-boronic acid, CuNPs, and H_2_O_2_. The reaction products are typically extracted with diethyl ether after the reaction [238].

#### 2.4.4. Mechanism of Degradation of Toxic Organic Dyes by Biogenically Synthesized CuNPs

**Figure 5:**
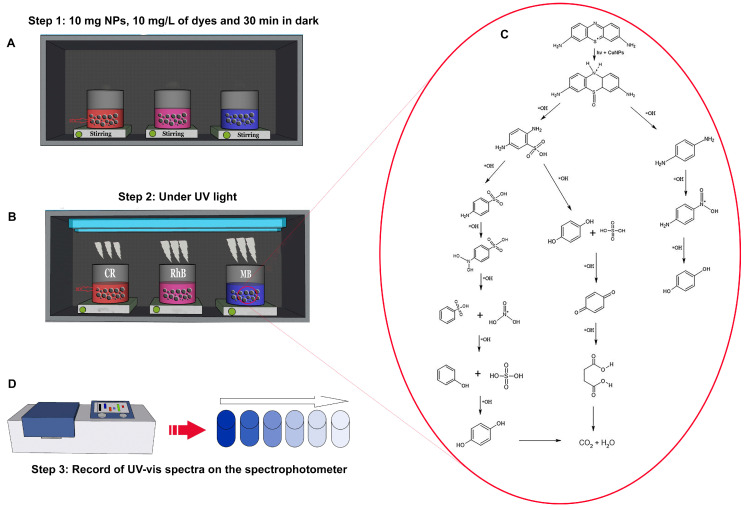
Photocatalytic activity of MB, RhB, and CR using CuNPs under similar conditions. (**A**) Step 1: degradation process in dark conditions, (**B**) step 2: degradation process under UV Light, (**C**) spectrophotometer UV-Vis, and (**D**) synthesis of MB. Step 3: spectrophotometer UV-Vis.

Figure 5 is an example of the degradation mechanism of toxic organic dyes and a compilation of previously studied articles. In this instance, three dyes were considered: methylene blue, rhodamine, and congo red [216]. In most of the articles, the three dyes had similar concentrations of 10 mg/L plus 10 mg of CuNPs. This mixture was stirred constantly for 30 min (Figure 5A). After, the solution was exposed to UV light and stirred continuously for 60 min (Figure 5B). Following these procedures, the sample was analyzed using a UV-visible spectrophotometer (Figure 5C), determining the percentage of dye degradation. The synthesis of methylene blue was also analyzed (Figure 5D). CuNPs degrade most dyes more efficiently than silver nanoparticles, particularly crystal violet [177]. Several studies have examined the significance of stirring the dye solution with the CuNPs in the dark for a few minutes to achieve the equilibrium of the adsorption and desorption of the dye with the nanoparticle surface before exposing them to sunlight or UV light, which degrades them rapidly [187].

As mentioned above, CuNPs generate electron–hole pairs via photon absorption, which is the basis for explaining the preceding technique. Electrons created in the valence band migrate to the hole in the conduction band. These valence band holes combine with hydroxyl ions to produce hydroxyl radicals (•OH). Superoxide radicals form when conduction band electrons react with dissolved oxygen. Superoxide radicals react with water to increase the concentration of •OH [239]. Due to their high oxidizing potential, •OH radicals degrade organic pollutants effectively. Bonds are broken, rings are opened, and oxidation occurs in degradation. Due to the substitution of •OH, polyhydroxylated products are easily removed from the benzene ring under radical attack [240] and break down into less hazardous fragments, such as NO_3_, SO_4_^2−^, CO_2_, and H_2_O [227].

Based on the presented data, it is strongly recommended to prioritize and intensify further research on the effect of plant extracts on the synthesis of CuNPs. Synthesis utilizing plant extracts is a sustainable and environmentally friendly method with promising applications. However, additional research is required to fully explore the potential of plant extracts since specific data on the proper effect or synergic interaction are explicitly reported. Plant extracts are a rich source of different bioactive compounds, and their synergistic interactions with CuNPs can enhance the activities under study. In addition, by examining the effect of plant extracts, specific antibacterial or antitumor properties can be identified, allowing for targeted applications against antibiotic-resistant strains and cancer cell lines. More research in this area will unlock the potential of plant extract-mediated synthesis, resulting in the development of effective, sustainable, and economically viable solutions for addressing bacterial infections and cancer cell line resistance.

## 3. Materials and Methods

### 3.1. Search Strategy

We systematically reviewed research articles published in English, excluding non-English publications, case reports, books, letters, and patents. We analyzed the research on CuNP biogenic or green synthesis and their primary applications.

We formulate the problem based on the premise that there is no systematic review of the principal applications of biogenically synthesized CuNPs.

A bibliographic search was conducted from 2012 to date, using the PubMed, Web of Science (WOS) Core Collection, and SCOPUS databases (September 2014 to January 2023). The results were deduplicated and uploaded to EndNote (Clarivate Analytics) and Rayyan Software. The preferred reporting items for systematic reviews and meta-analyses (PRISMA) recommendations were followed. The protocol for this systematic review was registered on the International Platform of Registered Systematic Review and Meta-analysis Protocols (INPLASY) (INPLASY202350109) and is available in full at inplasy.com (https://inplasy.com/inplasy-2023–5-0109/, accessed on 15 June 2023). The systematic review has been elaborated according to the PRISMA 2020 checklist (Table S5).

### 3.2. Search Criteria

We carried a screening set for the biogenic or green synthesis of CuNPs in the databases mentioned above, using the keywords in the title and abstract: (nanoparticle*) AND (biogenic) OR (green AND synthesis) AND (copper) OR (cu) OR (cu2o) OR (cuo).

### 3.3. Study Selection, Data Extraction, and Quality Assessment

Four authors selected the studies and reviewed the titles and abstracts of all published articles using the Rayyan software, based on the selected criteria and keywords. To compile research articles on biogenic synthesis, the patents, clinical trials, reviews, duplicates, and in vivo test-related studies and articles were excluded.

CMLJ, ACL, TEU, JCDC, and LDGM carried out the review process and management by extracting the authors’ and content’s raw data in accordance with a standard procedure.

After the initial database data collection, we screened and categorized the major applications using four screening sets. The first set contained antitumor terms, the second set contained antioxidant terms, the third set contained antibacterial terms, and the fourth set contained catalytic effect and dye removal terms.

The quality and risk of bias were evaluated based on the Cochrane Handbook for Systematic Interventions recommendations (http://www.handbook.cochrane.org, accessed on 12 December 2023). Independently, the authors evaluated the possibility of bias during the review process. At any stage of the reviewing process, disagreements among the authors were discussed and resolved.

The keywords for inclusion in the Rayyan Software were

1. For the antitumoral analysis, the following keywords in the title and abstract were used in the research fields: Green synthesis, Biogenic, Cancer, Anticarcinogenic, Antitumoral, and Cytotoxic; they were also used as keywords to collect data that might be under one of these terms;
2. For the antioxidant analysis, the following keywords in the title and abstract were used in the research fields: Green synthesis, Biogenic, Antioxidant, and Oxidative, and these were also used to collect data that might fall under one of these terms;
3. For the antibacterial analysis, the following keywords in the title and abstract were used in the research fields: Nanoparticles, Copper, Antibacterial, Green synthesis, antimicrobial, and biogenic, which were also used as keywords to collect data that might fall under one of these terms;
4. For the catalytic effect and dye removal analysis, the following keywords in the title and abstract were used in the research fields: Green synthesis, Catalytic, Photocatalytic, and Biogenic, which were also used as keywords to collect data that might fall under one of these terms.

A MeaSurement Tool to Assess Systematic Reviews (AMSTAR) was used to assess and evaluate the validity, quality, and reliability of the systematic review, as the content of each article was evaluated by following a set of criteria. The 11-item eligibility instrument was used to determine these scores, with each item in each article receiving a score of 1 or 0: Can’t Answer (CA) and Not Applicable (NA). The lowest score was 5, and the highest score was 8. (Table 5). The quality of research is proportional to the score: a score between 8 and 11: good quality; a score between 4 and 7: moderate quality; a score between 0 and 3: lower quality [38].

### 3.4. Data Analysis

**Figure 6:**
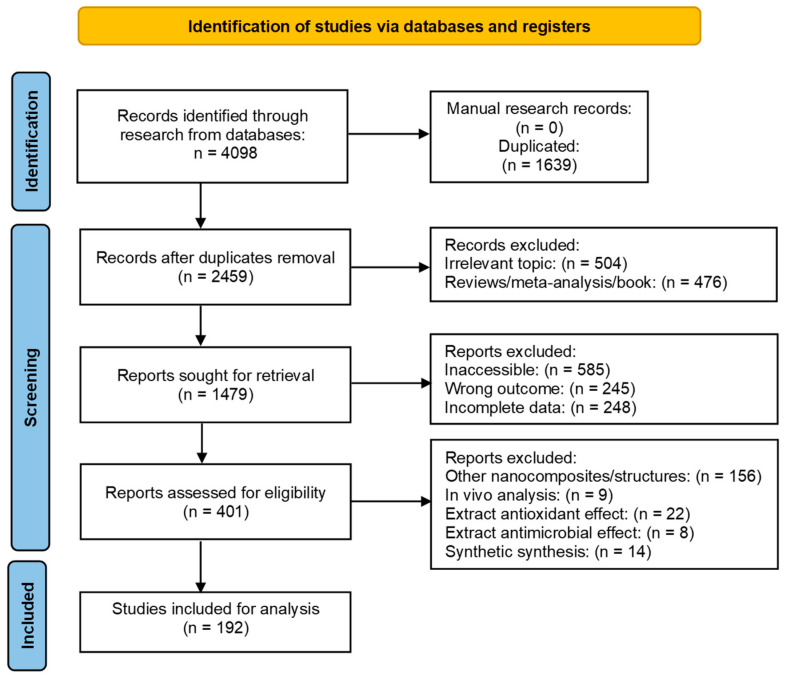
PRISMA (preferred reporting items for systematic reviews and meta-analysis).

During the systematic review, we collected qualitative data. Using the free VOSviewer server software (Version 1.6.15; https://www.vosviewer.com, accessed on 23 January 2023), we compiled, analyzed, and plotted the publication datasets, observing the trends and the most studied fields and applications on the biogenic synthesis of CuNPs. We established a threshold of 30 keyword co-occurrences. These co-occurrences were words that appeared in the analysis more than 29 times. The strategy process is summarized in Figure 6. It was unnecessary to conduct a meta-analysis because the current study did not reveal any discrepant findings for CuO.

## 4. Conclusions

The biogenic synthesis of CuNPs is a promising method for producing functional and stable nanoparticles with numerous potential applications. Biogenic synthesis has several advantages over conventional chemical synthesis methods, including low toxicity, cost-effectiveness, and mild reaction conditions. In addition, these nanoparticles have antioxidant, antibacterial, and antitumor properties, making them useful for healthcare and medical applications.

Biologically synthesized CuNPs have demonstrated catalytic potential in a variety of chemical reactions due to their large surface area and unique surface properties. As effective catalysts, they can enhance reaction rates and selectivity, and their production can support environmentally friendly and sustainable chemical industry processes. Due to their antioxidant and antitumor properties, the biogenic synthesis of CuNPs has shown great promise in medicine as antimicrobial agents and for cancer treatment and prevention.

The potential applications of biogenic CuNPs, which include antibacterial, antitumor, antioxidant, and catalytic properties, make them an exciting study area. However, there are still challenges that need to be overcome for the further advancement and utilization of CuNPs, such as optimizing the synthesis parameters, tailoring the properties for specific applications, ensuring biodegradability, achieving scalability, understanding the mechanisms of action, addressing safety concerns, and integrating CuNPs into emerging technologies. By overcoming these challenges through research, the full potential of CuNPs will be unlocked and expand their applications in diverse fields.

The integration of CuNPs with emerging technologies, such as nanomedicine and nanoelectronics, can create new diagnostics, therapeutics, and material applications. Collaboration between researchers, regulatory bodies, and industry stakeholders will be essential in establishing safety guidelines, standardizing protocols, and translating the potential of biogenic CuNPs into real-world applications, which will ultimately benefit sectors such as healthcare, catalysis, energy, and environmental remediation.

## Figures and Tables

**Table 1:** The antioxidant effect of biogenically synthetized CuNPs, salt, concentration, and biological source.

Plant	Salt Concentration (mM)	Part of the Plant	Results		Reference
			Antioxidant Activity (%) ^a^	IC50 (µg/mL)	
Copper acetate					
*Azadirachta indica*	200	Leaves	-	-	[19]
*Eclipta prostrata*	3	Leaves	53	-	[20]
*Monotheca buxifolia*	50	Leaves	53.40	-	[21]
*Borreria hispida*	10	Plant	-	0.6	[22]
*Cissus vitiginea*	-	Leaves	-	45.2	[23]
Copper nitrate					
*Eryngium caucasicum*	10	Leaves	40.02	-	[24]
*Juglans regia*	-	Leaves	78.80–90	-	[25]
*Solanum nigrum*	100	Leaves	90	-	[26]
*Galeopsidis herba*	-	Extract	-	4.12	[27]
*Allium noeanum*	0.1	Leaves	-	160	[28]
*Abutilon indicum*	-	Leaves	-	84	[29]
*Allium sativum*	100	Extract	-	40	[30]
*Plectranthus amboinicus*	100	Leaves	-	40.10	[31]
*Solanum nigrum*	10	Leaves	-	60	[26]
*Tinospora cordifolia*	-	Leaves	-	566	[32]
Copper sulfate					
*Ocimum basilicum*	100	Extract	-	-	[33]
*Sargassum longifolium*	10	Seaweed	-	-	[34]
*Thymbra spicata*	1	Leaves	-	-	[35]
*Berberis thunbergii*	200	Leaves	-	-	[17]
*Achillea nobilis*	100	Branch	44–48	-	[36]
*Prunus mahaleb*	50	Leaves	15.90	-	[37]
*Cissus vitiginea*	10	Leaves	21	-	[38]
*Persea americana*	-	Seed	23	-	[16]
*Mangifera indica*	3	Leaves	36.9	-	[39]
*Cissus arnotiana*	10	Leaves	21	-	[14]
*Protoparmeliopsis muralis*	100	Lichen	31.02	-	[18]
*Falcaria vulgaris*	40	Leaves	-	190	[40]
*Malus domestica*	1000	Leaves	-	45.90	[41]
*Cystoseira trinodis (algae)*	-	-	-	543	[42]
*Salvia hispanica*	100	Leaves	-	-	[43]
*Zingiber officinale*	280	Rhizome	81	-	[44]
*Moringa oleifera*		Leaves	60–70	-	[45]
*Artemisia haussknechtii*	100	Leaves	74.45	-	[46]
*Actin- omycetes (bacteria)*	20	-	-	-	[47]
*Actin- omycetes (bacteria)*	20	-	-	-	[47]
*Laurus nobilis*	1	Leaves	-	-	[48]
*Triticum aestivum*	40	Herb	-	-	[49]
*Pleurotus ostreatus*	2	Seed	-	-	[50]
*Urtica dioica*	10	Leaves	-	-	[51]
*Pleurotus ostreatus*	2	Biomass	-	-	[50]
Copper chloride					
*Koelreuteria paniculata*	10	Seeds	14.54	-	[52]
*Camellia sinensis*	250	Leaves	-	14	[53]

^a^ Percentage compared to standards antioxidants.

**Table 3:** The antibacterial effect of biogenically synthetized CuNPs, salt, concentration, and biological source.

Natural Extract Source	Part of the Plant	Size (nm)	Shape	NP Concentration	Micro-Organisms	Reference
Copper acetate						
*Sargassum swartzii*	Whole	32	Spherical	25 μg/mL	*V. parahaemolyticus*	[120]
*Averrhoa carambola*	Fruit	15	Spherical, square, and hexagonal	20 μg/mL	*S. aureus*, *Bacillius* spp., *Pseudomona* spp.	[118]
*Cylindrospermum stagnale*	Biomass	100	Spherical	8 mM	*C. albicans*, *K. pneumonia*, *E. cloacae*, *P. aeruginosa*, *E. coli*	[76]
*Penicillium chrysogenum*	Filtrate	10–190	Crystalline	50 μg/mL	*K. oxytoca*, *E. coli*, *S. aureus*, *B. cereus*	[121]
*Ocimum tenuiflorum*	Leaves	12–44	Spherical	3125–12,500 μg/mL	*B. subtilis*, *S. aureus*, *E.coli*	[87]
*Aloe-vera*	Leaves	45–95	Elliptical	1562 μg/mL	*B. subtilis*, *S. aureus*, *E.coli*	[122]
*Camellia Sinensis*	Leaves	25–32	Crystalline	1000 µg/mL	*S. aureus*, *B. subtilis*, *E. coli*, *K. pneumonia*	[123]
*Mimosa hamata*	Flower	40	-	0.1 g/mL	*E. coli* and *B. cereus*	[124]
*Polyalthia longifolia*	Leaves	50–60	Quasi-spherical	100–1000 µg/mL	*S. pyogenes*, *S. aureus*, *E. coli*, and *P. aeruginosa*, *E. floccosum*, *C. albicans*, *A. niger*, *A. clavatus*	[125]
*Botryococcus braunii*	Biomass	10–70	Spherical and cubical	250 µg/mL	*P. aeruginosa*, *E. coli*, *K. pneumoniae*, *S. aureus*, *F. oxysporum*	[126]
*Clematis orientalis*	Leaves	13–53	Crystalline	0.25 M	*S. aureus*, *B. subtilis*, *E. coli*, *P. aeruginosa*, *K. pneumoniae*	[127]
Copper nitrate						
*Allium sativum*	Root	20–40	Circular	150 μg/mL	*E. coli*, *S. aureus*, *B. subtilis*, *S. pyogenes*, *P. aeruginosa*, *K. pneumoniae*	[30]
*Zingiber officinale and Allium sativum*	Root	20–45	Spherical, agglomerated	1000 μg/mL	*MDR S. aureus*	[111]
*Tinospora cordifolia*	Leaves	10	Spherical	1001 μg/mL	*K. aerogenes*, *E. coli*, *P. desmolyticum*, *S. aureus*	[32]
*Withania somnifera*	Root	5–9	Spherical	100 μg/mL	*E. coli* and *S. aureus*	[128]
*Morus alba* L.	Fruit	50–200	Spherical and non-regular	2500 μg/mL	*E.coli* and *L. monocytogenes*	[129]
*Alpinia galangal*	Rhizome	20–60	Irregular spherical	10 mg/mL	*S. mutans*, *B. cereus*, *P. vulgaris*, *S. marcences*	[130]
*Cordia sebestena*	Flower	20–35	Spherical	75 µg/mL	*B. subtilis*, *S. aureus* and *E. coli*, *K. pneumoniae*	[131]
*Cassia fistula and Melia azedarach*	Leaves	43.8	Spherical	0.5 mg/mL	*K. pneumonia* and *H. pylori*	[132]
*Gloriosa superba* L.	Leaves	5–10	Spherical	1000 µg/mL	*K. aerogenes*, *P. desmolyticum*, *E. coli*, *S. aureus*	[133]
*Solanum nigrum*	Leaves	25	Spherical	100 μg/mL	*B. subtilis*, *S. saprohyticus*, *E. coli*, *P. aeruginosa*	[26]
*Rauvolfia serpentina*	Leaves	10–20	Crystalline	1000 μg/mL	*E. coli*, *P. desmolyticum*, *S. aureus*	[134]
*Aloe vera*	Leaves	20	Crystalline	100 μg/mL	*A. hydrophila*, *P. fluorescens*, *F. branchiophilum*	[135]
*Hibiscus cannabinus*	Leaves	10–40	Crystalline	5–31 mg/mL	*B. cereus*, *S. aureus*, *E. coli*, *K. pneumoniae*	[136]
*Capsicum frutescens*	Leaves	20–40	Spherical and rectangular	150 μg/mL	*K. pneumoniae*, *B. anthracis*, *L. monocytogenes*	[137]
*Eryngium caucasicum*	Leaves	40	Spherical	30–100 μg/mL	*E. coli*, *S. typhimurium*, *B. cereus* and *S. aureus*	[24]
*Menthe*	Biomass	22–25	Cubic, crystalline	250 µg/mL	*E. coli*, *B. subtilis*	[138]
*Saccharum officinarum*	Stem	5–140	Spherical	100 µg/mL	*E. coli*, *P. aeruginosa*, *S. aureus*, *B. subtilis*	[139]
*Catha edulis*	Leaves	28	Crystalline	40 mg/mL	*S. aureus*, *S. pyogenes*, *E. coli*, *K. pneumonia*	[140]
*Solanum tuberosum*	Tuber	54	Spherical	200–1000 µg/mL	*B. cereus*, *S sonnei*, *S. aureus*, *S. epidermidis*, *Enterococcus* spp., *P. aeruginosa*, *E. coli*	[141]
*Madhuca longifolia*	Flower/seeds	30–100	Spherical	10 mg/mL	*E.coli*, *B. subtilis*, *S. aureus*	[142]
*Opuntia ficus-indica*	Leaves	3–10	Spherical	100 µg/mL	*E. coli*	[143]
Copper sulfate						
*Falcaria vulgaris*	Leaf	20–25	Spherical	2 mg/mL	*S. pneumonia*, *B. subtilis*, *C. guilliermondii*, *C. krusei*	[40]
				4 mg/mL	*P. aeruginosa*, *S. aureus*, *C. albicans*, *C. glabrata*	
				8 mg/mL	*S. typhimurium*, *E. coli*	
				4 mg/mL	*S. pneumonia*, *B. subtilis*, *C. guilliermondii*, *C. kruse*	
				8 mg/mL	*P. aeruginosa*, *S. aureus*, *C. albicans*, *C. glabrata*	
				16 mg/mL	*S. typhimurium*, *E. coli*	
*Syzygium alternifolium*	Barks	50–100	Spherical	20 μg/mL	*B. subtilis*, *S. aureus*, *E.coli*, *K. pneumonia*, *P. vulgaris*, *P. aeruginosa*, *S. typhimurium*	[81]
					*A. solani*, *A. flavus*, *A. niger*, *P. chrysogenum*, *T. harzianum*	
				80 μg/mL	*Bacteria and fungi tested*	
*Cissus vitiginea*	Leaves	5–20	Spherical	75 μg/mL	*E. coli*, *Enterococcus* sp., *Proteus* sp., *Klebsiella* sp.	[38]
*Citrus Aurantifolia*	Leaves	10	Spherical-crystalline	50 μg/mL	*K. pneumoniae*, *S. aureus*	[102]
*Illicium verum*	Fruit	150–220	Undefined	100 μg/mL	*S. aureus*	[102]
*Myristica fragrans*	Fruit	210–270	Undefined	100 μg/mL	*S. aureus*	[102]
*Justicia gendarussa*	Leaves	50–100	Flower-shaped	75 μg/mL	*E. coli*, *S. aureus*	[144]
*Ruellia tuberosa*	Leaves	82	Spherical, cylindrical, and cubical	75 μg/mL	*S. aureus*, *K. pneumoniae*, *E. coli*	[145]
*Solanum lycopersicum*	Leaves	20–40	Spherical	300 μg/mL	*B. subtilis*, *S. aureus*, *E.coli*	[146]
*Sesbania aculeata*	Leaves	-	-	40 μg/mL	*P. destructive and C. lunata*	[147]
*Allium saralicum*	Leaves	45–50	Spherical	8 mg/mL	*C. albicans*, *C. glabrata*, *C. krusei*, *C. guilliermondii*, *P. aeruginosa*, *E.coli*, *B. subtilis*, *S. aureus*, *S. typhimurium*, *S. pneumoniae*	[148]
*Cystoseira myrica*, *Sargassum latifolium and Padina australis*	Leaves	40	Spherical	250 μg/mL	*E. coli* and *S. aureus*	[149]
*Tabernaemontana divaricate*	Leaves	46	Spherical	25 μg/mL	*E. coli*	[150]
*Acalypha indica*	Leaves	26	Spherical	25 μg/ml	*A. indica and C. albicans*	[86]
*Citrus medica*	Fruits	10–60	-	100 mM	*E. coli*, *P. acne*, *K. pneumoniae*, *S. typhi*, *and P. aeruginosa*, *F. oxysporum*, *F. graminearum* and *F. culmorum*	[151]
*Achillea Nobilis*	Flower	15–25	Hexagonal	50 μg/mL	*E. coli* and *S. aureus*	[36]
*Achyranthes aspera*	Leaves	95	Spherical	250 mM	*S. aureus* and *Gram-negative P. aeruginosa*	[152]
*Heliconia psittacorum*	Flower	12	Spherical	50 μg/mL	*S. aureus*, *P. putida*, *E. coli*	[153]
*Cedrus deodara*	Leaves	-	Spherical	150 μg/mL	*E. Coli*, *S. Aureus*, *S. Enterica*, *L. Monocytogenes*	[154]
*Sargassum longifolium*	Biomass	40–60	Spherical	100 μg/mL	*A. hydrophilla*, *V. harveyi*, *V. parahaemolyticus*, *S. marcescens*	[34]
*Cissus arnotiana*	Leaves	60–90	Spherical	50 μg/mL	*E. coli*, *Streptococcus* sp., *Rhizobium* sp., *Klebsiella* sp.	[14]
*Citrus aurantifolia*	Leaves	22	Crystalline	150 μg/mL	*S. aureus and E. coli*	[155]
*Pterolobium hexapetalum*	Leaves	10–50	Crystalline	50 µg/mL	*S. aureus*, *B. subtilis*, *E. coli*	[156]
*Convolvulus percicus* L.	Leaves	15–30	Crystalline	6.25 μg/mL	*S. aureus*, *E. coli*	[157]
*Cystoseira trinodis*	Biomass	6–7.8	Crystalline	10 μg/mL	*E.coli*, *E. faecalis*, *S. typhimurium*, *S. aureus*, *B. subtilis*, *S. faecalis*	[42]
*Azadirachta indica*	Flower	5	Spherical	40 μg/mL	*E. faecalis*, *P. mirabilis*, *K. pneumonia*, *S. aureus.*	[158]
*Mentha pulegium*	Leaves	21–48	Spherical	1000 μg/mL	*S. aureus*, *B. cereus*, *E.coli*, *K. pneumoniae*	[159]
*Brassica oleracea var. capitata f. rubra*	Leaves	77.5	Spherical	50 μg/mL	*E. coli*, *S. aureus*	[160]
*Passiflora foetida*		24.5	Crystalline	1000 µg/mL	*E.coli*, *S. typhimurium*, *A. aceti*	[161]
*Thymbra spicata*	Leaves	21–26.8	Spherical	100 µg/mL	*B. cereus*, *S. aureus*, *E.coli*, *S. typhimurium*	[35]
*Prunus mahaleb* L.	Whole	10–50	Spherical	0.5–2 mg/mL	*S. aureus*, *K. pneumonia*, *P. aeruginosa*, *E. coli*, *P. aeruginosa*	[37]
*Artemisia haussknechtii*	Leaves	35	Spherical	0.1 M	*E. coli*, *S. aureus*	[46]
Copper chloride						
*Tamarindus indica* L.	Fruit and leaves	50–100	Spherical	60 μL NP	*L. acidophilus*	[162]
				60 μL NP	*E. coli*	
				40 μL NP	*S. typhi*	
*Anacardium occidentale*	Shell	100		40 μg/mL	*B. linens*, *P. acnes*, *B. cereus*, *S. epidermidis*	[163]
*Ficus carica*	Leaves	41.5	Spherical	200 μg/mL	*Candida spp.*, *Aspergillus* spp., *S aureus*, *A. baumanii*	[164]
*Tinospora cardifolia*	Leaves	63–143	Spherical	175 μg/mL	*S. aureus and E. coli*	[165]
*Vitex negundo*	Root	40–60	Spherical, cubic and hexagonal	500 μg/mL	*B. subtilis*, *S. aureus*, *E. coli*, *P. aeruginosa*	[166]
*Cardiospermum halicacabum*	Leaves	40	Hexagonal	50 µg/mL	*P. aeruginosa*, *E. coli*, *S. aureus*	[98]
*Aspergillus niger*	Biomass	23–199	Crystalline	2.5 mg/mL	*P. aeruginosa*, *E. faecalis*, *E. coli*, *K. pneumonia*, *P. vulgari*, *S. aureus*, *C. albicans*, *A. niger*	[167]
*Brassica oleracea*, *Solanum tuberosum*, *Pisum sativum*	Peels	32.5, 40.75, 47.2	Spherical and cubical	45 µg/mL	*P. aeruginosa*, *E. coli*, *B. subtilus*, *S. aureus*	[168]
*Vaccinium myrtillus* L.	Fruit	2–10	Spherical	3.125 mM	*E. coli*, *C. albicans*, *S. cerevisiae*	[169]

**Table 5:** AMSTAR criteria table.

N°	Item
1	Was an ‘‘a priori’’ design provided?
2	Was there duplicate study selection and data extraction?
3	Was a comprehensive literature search performed?
4	Was the status of publication (i.e., grey literature) used as an inclusion criterion?
5	Was a list of studies (included and excluded) provided?
6	Were the characteristics of the included studies provided?
7	Was the scientific quality of the included studies assessed and documented?
8	Was the scientific quality of the included studies used appropriately in formulating the conclusions?
9	Were the methods used to combine the findings of studies appropriate?
10	Was the likelihood of publication bias assessed?
11	Were potential conflicts of interest included?

## Data Availability

Not applicable.

## Supplementary Materials

The following supporting information can be downloaded at: https://www.mdpi.com/article/10.3390/molecules28124838/s1, Table S1. AMSTAR table for the antioxidant effect analysis., Table S2. AMSTAR table for the antitumoral effect analysis., Table S3. AMSTAR table for the antibacterial effect analysis., Table S4. AMSTAR table for the catalytic/dye removal effect analysis., Table S5. PRISMA 2020 checklist. References [241,242] can be found in the Supplementary Materials.
