# Supplementary material for: Biogenic Synthesis of Copper Nanoparticles: A Systematic Review of Their Features and Main Applications
Source: Molecules. 2023 Jun 18;28(12):4838. doi: 10.3390/molecules28124838 (PMC10301071; doi:10.3390/molecules28124838)
Supplement: Supplementary file 1 [file molecules-28-04838-s001.zip › molecules-2445797-supplementary.pdf]

Supplementary Materials

Table S1. AMSTAR table for the antioxidant effect analysis.

**Quality Assesment Tool AMSTAR**													
**Citation**		**1**	**2**	**3**	**4**	**5**	**6**	**7**	**8**	**9**	**10**	**11**	**Score**
1	[1]	1	1	1	1	1	1	NA	0	NA	CA	1	7
2	[2]	1	1	1	1	1	1	NA	1	NA	CA	1	8
3	[3]	1	1	1	1	1	1	NA	1	NA	CA	1	8
4	[4]	1	1	1	1	1	1	NA	1	NA	CA	1	8
5	[5]	1	1	1	1	1	1	NA	1	NA	CA	1	8
6	[6]	1	1	1	1	1	1	NA	0	NA	CA	1	7
7	[7]	1	1	1	1	1	1	NA	1	NA	CA	1	8
8	[8]	1	1	1	1	1	1	NA	1	NA	CA	1	8
9	[9]	1	1	1	1	1	1	NA	1	NA	CA	1	8
10	[10]	1	1	1	1	1	1	NA	1	NA	CA	1	8
11	[11]	1	1	1	1	1	1	NA	1	NA	CA	1	8
12	[12]	1	1	1	1	1	1	NA	0	NA	CA	1	7
13	[13]	1	1	1	1	1	1	NA	0	NA	CA	1	7
14	[14]	1	1	1	1	1	1	NA	0	NA	CA	1	7
15	[15]	1	1	1	1	1	1	NA	1	NA	CA	1	8
16	[16]	1	1	1	1	1	1	NA	1	NA	CA	1	8
17	[17]	1	1	1	1	1	1	NA	0	NA	CA	1	7
18	[18]	1	1	1	1	1	1	NA	1	NA	CA	1	8
19	[19]	1	1	1	1	1	1	NA	1	NA	CA	1	8
20	[20]	1	1	1	1	1	1	NA	1	NA	CA	1	8

21	[21]	1	1	1	1	1	1	NA	1	NA	CA	1	8
22	[22]	1	1	1	1	1	1	NA	0	NA	CA	1	7
23	[23]	1	1	1	1	1	1	NA	0	NA	CA	1	7
24	[24]	1	1	1	1	1	1	NA	0	NA	CA	1	7
25	[25]	1	1	1	1	1	1	NA	1	NA	CA	1	8
26	[26]	1	1	1	1	1	1	NA	1	NA	CA	1	8
27	[27]	1	1	1	1	1	1	NA	1	NA	CA	1	8
28	[28]	1	1	1	1	1	1	NA	0	NA	CA	1	7
29	[29]	1	1	1	1	1	1	NA	1	NA	CA	1	8
30	[30]	1	1	1	1	1	1	NA	1	NA	CA	1	8
31	[31]	1	1	1	1	1	1	NA	0	NA	CA	1	7
32	[32]	1	1	1	1	1	1	NA	1	NA	CA	1	8
33	[33]	1	1	1	1	1	1	NA	1	NA	CA	1	8
34	[34]	1	1	1	1	1	1	NA	1	NA	CA	1	8
35	[35]	1	1	1	1	1	1	NA	1	NA	CA	1	8
36	[36]	1	1	1	1	1	1	NA	1	NA	CA	1	8
37	[37]	1	1	1	1	1	1	NA	1	NA	CA	1	8
38	[38]	1	1	1	1	1	1	NA	1	NA	CA	1	8
39	[39]	1	1	1	1	1	1	NA	1	NA	CA	1	8

Table S2. AMSTAR table for the antitumoral effect analysis.

**Quality Assessment Tool AMSTAR**													
**CITATION**		**1**	**2**	**3**	**4**	**5**	**6**	**7**	**8**	**9**	**10**	**11**	**SCORE**
1	[40]	1	1	1	0	1	1	NA	1	NA	CA	1	7
2	[41]	1	1	1	0	1	1	NA	1	NA	CA	1	7

3	[42]	1	1	1	0	1	1	NA	1	NA	CA	1	7
4	[43]	1	1	1	0	1	1	NA	1	NA	1	1	8
5	[44]	1	1	1	0	1	1	NA	1	NA	1	1	8
6	[45]	1	1	1	0	1	1	NA	1	NA	1	1	8
7	[7]	1	1	1	0	1	1	NA	1	NA	CA	1	7
8	[8]	1	1	1	0	1	1	NA	0	NA	1	1	7
9	[11]	1	1	1	0	1	1	NA	1	NA	CA	1	7
10	[46]	1	1	1	0	1	1	NA	1	NA	CA	1	7
11	[47]	1	1	1	0	1	1	NA	1	NA	CA	1	7
12	[48]	1	1	1	0	1	1	NA	0	NA	CA	1	6
13	[49]	1	1	1	0	1	1	NA	1	NA	1	1	8
14	[50]	1	1	1	0	1	1	NA	1	NA	1	1	8
15	[51]	1	1	1	0	1	1	NA	1	NA	CA	1	7
16	[52]	1	1	1	0	1	1	NA	1	NA	CA	1	7
17	[53]	1	1	1	0	1	1	NA	1	NA	CA	1	7
18	[54]	1	1	1	0	1	1	NA	1	NA	CA	1	7
19	[55]	1	1	1	0	1	1	NA	1	NA	CA	1	7
20	[56]	1	1	1	0	1	1	NA	1	NA	CA	1	7
21	[57]	1	1	1	0	1	1	NA	1	NA	CA	1	7
22	[58]	1	1	1	0	1	1	NA	1	NA	1	1	8
23	[59]	1	1	1	0	1	1	NA	1	NA	1	1	8
24	[60]	1	1	1	0	1	1	NA	1	NA	CA	1	7
25	[61]	1	1	1	0	1	1	NA	0	NA	1	1	7
26	[62]	1	1	1	0	1	1	NA	0	NA	1	1	7
27	[63]	1	1	1	0	1	1	NA	1	NA	1	1	8

28	[64]	1	1	1	0	1	1	NA	0	NA	CA	1	6
29	[65]	1	1	1	0	1	1	NA	1	NA	1	1	8
30	[66]	1	1	1	0	1	1	NA	0	NA	CA	1	6
31	[67]	1	1	1	0	1	1	NA	0	NA	CA	1	6
32	[31]	1	1	1	0	1	1	NA	1	NA	CA	1	7
33	[68]	1	1	1	0	1	1	NA	1	NA	1	1	8
34	[69]	1	1	1	0	1	1	NA	1	NA	CA	1	7
35	[70]	1	1	1	0	1	1	NA	1	NA	CA	1	7
36	[71]	1	1	1	0	1	1	NA	1	NA	CA	1	7
37	[72]	1	1	1	0	1	1	NA	1	NA	CA	1	7
38	[73]	1	1	1	0	1	1	NA	1	NA	CA	1	7
39	[74]	1	1	1	0	1	1	NA	0	NA	CA	1	6
40	[75]	1	1	1	0	1	1	NA	1	NA	1	1	8
41	[76]	1	1	1	0	1	1	NA	1	NA	CA	1	7
42	[77]	1	1	1	0	1	1	NA	1	NA	CA	1	7
43	[78]	1	1	1	0	1	1	NA	0	NA	CA	1	6
44	[79]	1	1	1	0	1	1	NA	1	NA	CA	1	7
45	[80]	1	1	1	0	1	1	NA	1	NA	CA	1	7
46	[81]	1	1	1	0	1	1	NA	1	NA	1	1	8

Table S3. AMSTAR table for the antibacterial effect analysis.

**Quality Assessment Tool AMSTAR**													
**Citation**		**1**	**2**	**3**	**4**	**5**	**6**	**7**	**8**	**9**	**10**	**11**	**Score**
1	[82]	1	1	1	0	1	1	NA	1	NA	CA	1	7
2	[83]	1	1	1	0	1	1	NA	1	NA	CA	1	7

3	[74]	1	1	1	0	1	1	NA	1	NA	CA	1	7
4	[84]	1	1	1	0	1	1	NA	1	NA	CA	1	7
5	[85]	1	1	1	1	1	1	NA	1	NA	CA	0	7
6	[86]	1	1	1	0	1	1	NA	1	NA	CA	1	7
7	[55]	1	1	1	0	1	1	NA	1	NA	CA	1	7
8	[87]	1	1	1	0	1	1	NA	1	NA	CA	1	7
9	[88]	1	1	1	1	1	1	NA	0	NA	CA	1	7
10	[89]	1	1	1	1	1	1	NA	1	NA	CA	1	8
11	[90]	1	1	1	1	1	1	NA	1	NA	CA	1	8
12	[35]	1	1	1	1	1	1	NA	1	NA	CA	1	8
13	[91]	1	1	1	1	1	1	NA	1	NA	CA	1	8
14	[34]	1	1	1	1	1	1	NA	1	NA	CA	1	8
15	[92]	1	1	1	1	1	1	NA	0	NA	CA	1	7
16	[93]	1	1	1	1	1	1	NA	1	NA	CA	1	8
17	[94]	1	1	1	0	1	1	NA	1	NA	CA	1	7
18	[95]	1	1	1	0	1	1	NA	1	NA	CA	1	7
19	[96]	1	1	1	0	1	1	NA	1	NA	CA	1	7
20	[97]	1	1	1	0	1	1	NA	1	NA	CA	1	7
21	[25]	1	1	1	0	1	1	NA	1	NA	CA	1	7
22	[98]	1	1	1	0	1	1	NA	1	NA	CA	1	7
23	[99]	1	1	1	0	1	1	NA	1	NA	CA	1	7
24	[100]	1	1	1	0	1	1	NA	1	NA	CA	1	7
25	[101]	1	1	1	0	1	1	NA	1	NA	CA	1	7
26	[16]	1	1	1	0	1	1	NA	1	NA	CA	1	7
27	[102]	1	1	1	0	1	1	NA	0	NA	CA	1	6

28	[103]	1	1	1	0	1	1	NA	1	NA	1	1	8
29	[104]	1	1	1	0	1	1	NA	1	NA	CA	1	7
30	[105]	1	1	1	0	1	1	NA	1	NA	CA	1	7
31	[106]	1	1	1	0	1	1	NA	1	NA	CA	1	7
32	[107]	1	1	1	0	1	1	NA	1	NA	CA	1	7
33	[39]	1	1	1	0	1	1	NA	1	NA	CA	1	7
34	[79]	1	1	1	0	1	1	NA	1	NA	CA	1	7
35	[38]	1	1	1	0	1	1	NA	1	NA	CA	1	7
36	[77]	1	1	1	0	1	1	NA	1	NA	CA	1	7
37	[108]	1	1	1	0	1	1	NA	1	NA	CA	1	7
38	[109]	1	1	1	0	1	1	NA	0	NA	CA	1	6
39	[110]	1	1	1	1	1	1	NA	1	NA	CA	1	8
40	[111]	1	1	1	1	1	1	NA	1	NA	CA	1	8
41	[112]	1	1	1	1	1	1	NA	1	NA	CA	1	8
42	[113]	1	1	1	1	1	1	NA	1	NA	CA	1	8
43	[114]	1	1	1	1	1	1	NA	1	NA	CA	1	8
44	[73]	1	1	1	1	1	1	NA	0	NA	CA	1	7
45	[115]	1	1	1	1	1	1	NA	0	NA	CA	1	7
46	[32]	1	1	1	1	1	1	NA	0	NA	CA	1	7
47	[116]	1	1	1	1	1	1	NA	1	NA	CA	1	8
48	[117]	1	1	1	1	1	1	NA	1	NA	CA	1	8
49	[118]	1	1	1	1	1	1	NA	0	NA	CA	1	7
50	[28]	1	1	1	0	1	1	NA	1	NA	CA	1	7
51	[27]	1	1	1	0	1	1	NA	1	NA	CA	1	7
52	[119]	1	1	1	0	1	1	NA	1	NA	CA	1	7

53	[120]	1	1	1	0	1	1	NA	1	NA	CA	1	7
54	[121]	1	1	1	0	1	1	NA	0	NA	1	1	7
55	[15]	1	1	1	0	1	1	NA	1	NA	CA	1	7
56	[122]	1	1	1	0	1	1	NA	1	NA	1	1	8
57	[123]	1	1	1	0	1	1	NA	1	NA	1	1	8
58	[124]	1	1	1	0	1	1	NA	1	NA	CA	1	7
59	[125]	1	1	1	0	1	1	NA	1	NA	1	1	8
60	[13]	1	1	1	0	1	1	NA	1	NA	CA	1	7
61	[9]	1	1	1	0	1	1	NA	1	NA	CA	1	7
62	[3]	1	1	1	1	1	1	NA	1	NA	CA	1	8
63	[126]	1	1	1	1	1	1	NA	1	NA	CA	1	8
64	[127]	1	1	1	1	1	1	NA	1	NA	CA	1	8
65	[128]	1	1	1	1	1	1	NA	1	NA	CA	1	8
66	[129]	1	1	1	1	1	1	NA	1	NA	CA	1	8
67	[130]	1	1	1	1	1	1	NA	0	NA	CA	1	7
68	[61]	1	1	1	1	1	1	NA	0	NA	CA	1	7
69	[131]	1	1	1	1	1	1	NA	0	NA	CA	1	7
70	[132]	1	1	1	1	1	1	NA	1	NA	CA	1	8
71	[133]	1	1	1	1	1	1	NA	1	NA	CA	1	8

Table S4. AMSTAR table for the catalytic/dye removal effect analysis.

Quality Assessment Tool AMSTAR													
**Citation**		**1**	**2**	**3**	**4**	**5**	**6**	**7**	**8**	**9**	**10**	**11**	**Score**
1	[134]	1	1	1	0	1	1	NA	1	NA	CA	1	7
2	[135]	1	1	1	0	1	1	NA	1	NA	CA	1	7
3	[136]	1	1	1	0	1	1	NA	1	NA	CA	1	7

4	[56]	1	1	1	0	1	1	NA	1	NA	CA	1	7
5	[137]	1	1	1	0	1	1	NA	0	NA	CA	0	5
6	[138]	1	1	1	0	1	1	NA	1	NA	CA	1	7
7	[15]	1	1	1	0	1	1	NA	1	NA	CA	1	7
8	[139]	1	1	1	0	1	1	NA	1	NA	CA	1	7
9	[140]	1	1	1	0	1	1	NA	1	NA	CA	1	7
10	[141]	1	1	1	0	1	1	NA	1	NA	CA	1	7
11	[142]	1	1	1	0	1	1	NA	1	NA	CA	1	7
12	[85]	1	1	1	0	1	1	NA	0	NA	CA	1	6
13	[143]	1	1	1	0	1	1	NA	1	NA	CA	1	7
14	[144]	1	1	1	0	1	1	NA	1	NA	CA	1	7
15	[121]	1	1	1	0	1	1	NA	1	NA	CA	1	7
16	[145]	1	1	1	0	1	1	NA	1	NA	CA	1	7
17	[146]	1	1	1	0	1	1	NA	1	NA	CA	1	7
18	[14]	1	1	1	0	1	1	NA	1	NA	CA	1	7
18	[147]	1	1	1	0	1	1	NA	1	NA	CA	1	7
20	[148]	1	1	1	0	1	1	NA	1	NA	CA	1	7
21	[149]	1	1	1	0	1	1	NA	1	NA	CA	1	7
22	[150]	1	1	1	0	1	1	NA	1	NA	CA	1	7
23	[151]	1	1	1	0	1	1	NA	1	NA	CA	1	7
24	[152]	1	1	1	0	1	1	NA	1	NA	CA	1	7
25	[95]	1	1	1	0	1	1	NA	1	NA	CA	1	7
26	[25]	1	1	1	0	1	1	NA	1	NA	CA	1	7
27	[110]	1	1	1	0	1	1	NA	1	NA	CA	1	7
28	[153]	1	1	1	0	1	1	NA	0	NA	CA	1	6

29	[154]	1	1	1	0	1	1	NA	1	NA	1	1	8
30	[155]	1	1	1	0	1	1	NA	1	NA	CA	1	7
31	[156]	1	1	1	0	1	1	NA	1	NA	CA	1	7
32	[157]	1	1	1	0	1	1	NA	1	NA	CA	1	7
33	[11]	1	1	1	0	1	1	NA	1	NA	CA	1	7
34	[120]	1	1	1	0	1	1	NA	1	NA	CA	1	7
35	[158]	1	1	1	0	1	1	NA	1	NA	CA	1	7
36	[159]	1	1	1	0	1	1	NA	1	NA	CA	1	7
37	[160]	1	1	1	0	1	1	NA	1	NA	CA	1	7
38	[161]	1	1	1	0	1	1	NA	1	NA	CA	1	7
39	[162]	1	1	1	0	1	1	NA	1	NA	CA	1	7
40	[119]	1	1	1	0	1	1	NA	0	NA	CA	1	6
41	[163]	1	1	1	0	1	1	NA	1	NA	CA	1	7
42	[61]	1	1	1	0	1	1	NA	1	NA	CA	1	7
43	[164]	1	1	1	0	1	1	NA	1	NA	CA	1	7
44	[165]	1	1	1	0	1	1	NA	1	NA	CA	1	7
45	[166]	1	1	1	0	1	1	NA	1	NA	CA	1	7
46	[167]	1	1	1	0	1	1	NA	1	NA	CA	1	7
47	[168]	1	1	1	0	1	1	NA	1	NA	CA	1	7
48	[169]	1	1	1	0	1	1	NA	1	NA	CA	1	7
49	[129]	1	1	1	0	1	1	NA	0	NA	CA	1	6
50	[170]	1	1	1	0	1	1	NA	0	NA	CA	1	6
51	[171]	1	1	1	0	1	1	NA	1	NA	CA	1	7
52	[172]	1	1	1	0	1	1	NA	1	NA	CA	1	7
53	[173]	1	1	1	0	1	1	NA	1	NA	CA	1	7

54	[174]	1	1	1	0	1	1	NA	1	NA	CA	1	7
55	[175]	1	1	1	0	1	1	NA	1	NA	CA	1	7
56	[176]	1	1	1	0	1	1	NA	1	NA	CA	1	7
57	[177]	1	1	1	0	1	1	NA	1	NA	CA	1	7
58	[98]	1	1	1	0	1	1	NA	1	NA	CA	1	7
59	[178]	1	1	1	0	1	1	NA	0	NA	CA	0	5
60	[179]	1	1	1	0	1	1	NA	1	NA	CA	1	7
61	[180]	1	1	1	0	1	1	NA	1	NA	CA	1	7
62	[181]	1	1	1	0	1	1	NA	1	NA	CA	1	7
63	[182]	1	1	1	0	1	1	NA	1	NA	CA	1	7
64	[183]	1	1	1	0	1	1	NA	1	NA	CA	1	7
65	[184]	1	1	1	0	1	1	NA	0	NA	1	1	7
66	[185]	1	1	1	0	1	1	NA	1	NA	CA	1	7
67	[186]	1	1	1	0	1	1	NA	1	NA	1	1	8
68	[187]	1	1	1	0	1	1	NA	1	NA	1	1	8
69	[188]	1	1	1	0	1	1	NA	1	NA	CA	1	7
70	[189]	1	1	1	0	1	1	NA	1	NA	1	1	8
71	[190]	1	1	1	0	1	1	NA	1	NA	CA	1	7
72	[191]	1	1	1	0	1	1	NA	1	NA	CA	1	7
73	[192]	1	1	1	0	1	1	NA	0	NA	CA	1	6

Table S5. PRISMA Checklist.

Section and Topic	Item #	Checklist item	Location where item is reported
**TITLE**			
Title	1	Identify the report as a systematic review.	1
**ABSTRACT**			
Abstract	2	See the PRISMA 2020 for Abstracts checklist.	1
**INTRODUCTION**			
Rationale	3	Describe the rationale for the review in the context of existing knowledge.	2
Objectives	4	Provide an explicit statement of the objective(s) or question(s) the review addresses.	2
**METHODS**			
Eligibility criteria	5	Specify the inclusion and exclusion criteria for the review and how studies were grouped for the syntheses.	30
Information sources	6	Specify all databases, registers, websites, organisations, reference lists and other sources searched or consulted to identify studies. Specify the date when each source was last searched or consulted.	30
Search strategy	7	Present the full search strategies for all databases, registers and websites, including any filters and limits used.	30
Selection process	8	Specify the methods used to decide whether a study met the inclusion criteria of the review, including how many reviewers screened each record and each report retrieved, whether they worked independently, and if applicable, details of automation tools used in the process.	30-31
Data collection process	9	Specify the methods used to collect data from reports, including how many reviewers collected data from each report, whether they worked independently, any processes for obtaining or confirming data from study investigators, and if applicable, details of automation tools used in the process.	30-31
Data items	10a	List and define all outcomes for which data were sought. Specify whether all results that were compatible with each outcome domain in each study were sought (e.g. for all measures, time points, analyses), and if not, the methods used to decide which results to collect.	30-31
	10b	List and define all other variables for which data were sought (e.g. participant and intervention characteristics, funding sources). Describe any assumptions made about any missing or unclear information.	30-31
Study risk of bias assessment	11	Specify the methods used to assess risk of bias in the included studies, including details of the tool(s) used, how many reviewers assessed each study and whether they worked independently, and if applicable, details of automation tools used in the process.	30-31
Effect measures	12	Specify for each outcome the effect measure(s) (e.g. risk ratio, mean difference) used in the synthesis or presentation of results.	N/A
Synthesis methods	13a	Describe the processes used to decide which studies were eligible for each synthesis (e.g. tabulating the study intervention characteristics and comparing against the planned groups for each synthesis (item #5)).	30-31
	13b	Describe any methods required to prepare the data for presentation or synthesis, such as handling of missing summary statistics, or data	30-31

Section and Topic	Item #	Checklist item	Location where item is reported
		conversions.	
	13c	Describe any methods used to tabulate or visually display results of individual studies and syntheses.	31
	13d	Describe any methods used to synthesize results and provide a rationale for the choice(s). If meta-analysis was performed, describe the model(s), method(s) to identify the presence and extent of statistical heterogeneity, and software package(s) used.	30
	13e	Describe any methods used to explore possible causes of heterogeneity among study results (e.g. subgroup analysis, meta-regression).	N/A
	13f	Describe any sensitivity analyses conducted to assess robustness of the synthesized results.	N/A
Reporting bias assessment	14	Describe any methods used to assess risk of bias due to missing results in a synthesis (arising from reporting biases).	30-31
Certainty assessment	15	Describe any methods used to assess certainty (or confidence) in the body of evidence for an outcome.	N/A
**RESULTS**			
Study selection	16a	Describe the results of the search and selection process, from the number of records identified in the search to the number of studies included in the review, ideally using a flow diagram.	32
	16b	Cite studies that might appear to meet the inclusion criteria, but which were excluded, and explain why they were excluded.	32
Study characteristics	17	Cite each included study and present its characteristics.	3-29
Risk of bias in studies	18	Present assessments of risk of bias for each included study.	30-31
Results of individual studies	19	For all outcomes, present, for each study: (a) summary statistics for each group (where appropriate) and (b) an effect estimate and its precision (e.g. confidence/credible interval), ideally using structured tables or plots.	5, 10,16, 23
Results of syntheses	20a	For each synthesis, briefly summarise the characteristics and risk of bias among contributing studies.	3-32, Tables S1-S4
	20b	Present results of all statistical syntheses conducted. If meta-analysis was done, present for each the summary estimate and its precision (e.g. confidence/credible interval) and measures of statistical heterogeneity. If comparing groups, describe the direction of the effect.	N/A
	20c	Present results of all investigations of possible causes of heterogeneity among study results.	3-32, Tables S1-S4
	20d	Present results of all sensitivity analyses conducted to assess the robustness of the synthesized results.	N/A
Reporting biases	21	Present assessments of risk of bias due to missing results (arising from reporting biases) for each synthesis assessed.	N/A

Section and Topic	Item #	Checklist item	Location where item is reported
Certainty of evidence	22	Present assessments of certainty (or confidence) in the body of evidence for each outcome assessed.	N/A
**DISCUSSION**			
Discussion	23a	Provide a general interpretation of the results in the context of other evidence.	3-29
	23b	Discuss any limitations of the evidence included in the review.	3-29
	23c	Discuss any limitations of the review processes used.	3-29
	23d	Discuss implications of the results for practice, policy, and future research.	32
**OTHER INFORMATION**			
Registration and protocol	24a	Provide registration information for the review, including register name and registration number, or state that the review was not registered.	30
	24b	Indicate where the review protocol can be accessed, or state that a protocol was not prepared.	30
	24c	Describe and explain any amendments to information provided at registration or in the protocol.	30
Support	25	Describe sources of financial or non-financial support for the review, and the role of the funders or sponsors in the review.	33
Competing interests	26	Declare any competing interests of review authors.	33
Availability of data, code and other materials	27	Report which of the following are publicly available and where they can be found: template data collection forms; data extracted from included studies; data used for all analyses; analytic code; any other materials used in the review.	30

From: Page MJ, McKenzie JE, Bossuyt PM, Boutron I, Hoffmann TC, Mulrow CD, et al. The PRISMA 2020 statement: an updated guideline for reporting systematic reviews. BMJ 2021;372:n71. doi: 10.1136/bmj.n71

For more information, visit: http://www.prisma-statement.org/

References

1. Abbasifar, A.; Shahrabadi, F.; ValizadehKaji, B. Effects of Green Synthesized Zinc and Copper Nano-Fertilizers on the Morphological and Biochemical Attributes of Basil Plant. *J Plant Nutr* **2020**, *43*, 1104–1118, doi:10.1080/01904167.2020.1724305.

2. Alavi, M.; Karimi, N.; Valadbeigi, T. Antibacterial, Antibiofilm, Antiquorum Sensing, Antimotility, and Antioxidant Activities of Green Fabricated Ag, Cu, TiO2, ZnO, and Fe3O4 NPs via Protoparmeliopsis Muralis Lichen Aqueous Extract against Multi-Drug-Resistant Bacteria. *ACS Biomater Sci Eng* **2019**, *5*, 4228–4243, doi:10.1021/acsbiomaterials.9b00274.

3. Alavi, M.; Karimi, N. Characterization, Antibacterial, Total Antioxidant, Scavenging, Reducing Power and Ion Chelating Activities of Green Synthesized Silver, Copper and Titanium Dioxide Nanoparticles Using Artemisia Haussknechtii Leaf Extract. *Artif Cells Nanomed Biotechnol* **2018**, *46*, 2066–2081, doi:10.1080/21691401.2017.1408121.

4. Ali, J.S.; Mannan, A.; Nasrullah, M.; Ishtiaq, H.; Naz, S.; Zia, M. Antimicrobial, Antioxidative, and Cytotoxic Properties of Monotheca Buxifolia Assisted Synthesized Metal and Metal Oxide Nanoparticles. *Inorganic and Nano-Metal Chemistry* **2020**, *50*, 770–782, doi:10.1080/24701556.2020.1724150.

5. Asemani, M.; Anarjan, N. Green Synthesis of Copper Oxide Nanoparticles Using Juglans Regia Leaf Extract and Assessment of Their Physico-Chemical and Biological Properties. *Green Processing and Synthesis* **2019**, *8*, 557–567, doi:10.1515/gps-2019-0025.

6. Bulut Kocabas, B.; Attar, A.; Peksel, A.; Altikatoglu Yapaoz, M. Phytosynthesis of CuONPs via Laurus Nobilis: Determination of Antioxidant Content, Antibacterial Activity, and Dye Decolorization Potential. *Biotechnol Appl Biochem* **2021**, *68*, 889–895, doi:10.1002/bab.2010.

7. Chinnathambi, A.; Awad Alahmadi, T.; Ali Alharbi, S. Biogenesis of Copper Nanoparticles (Cu-NPs) Using Leaf Extract of Allium Noeanum, Antioxidant and in-Vitro Cytotoxicity. *Artif Cells Nanomed Biotechnol* **2021**, *49*, 500–510, doi:10.1080/21691401.2021.1926275.

8. Chung, I.; Rahuman, A.A.; Marimuthu, S.; Kirthi, A. V; Anbarasan, K.; Padmini, P.; Rajakumar, G. Green Synthesis of Copper Nanoparticles Using Eclipta Prostrata Leaves Extract and Their Antioxidant and Cytotoxic Activities. *Exp Ther Med* **2017**, *14*, 18–24, doi:10.3892/etm.2017.4466.

9. Dashtizadeh, Z.; Jookar Kashi, F.; Ashrafi, M. Phytosynthesis of Copper Nanoparticles Using Prunus Mahaleb L. and Its Biological Activity. *Mater Today Commun* **2021**, *27*, doi:10.1016/j.mtcomm.2021.102456.

10. Dobrucka, R. Antioxidant and Catalytic Activity of Biosynthesized CuO Nanoparticles Using Extract of Galeopsidis Herba. *J Inorg Organomet Polym Mater* **2018**, *28*, 812–819, doi:10.1007/s10904-017-0750-2.

11. Dou, L.; Zhang, X.; Zangeneh, M.M.; Zhang, Y. Efficient Biogenesis of Cu2O Nanoparticles Using Extract of Camellia Sinensis Leaf: Evaluation of Catalytic, Cytotoxicity, Antioxidant, and Anti-Human Ovarian Cancer Properties. *Bioorg Chem* **2021**, *106*, doi:10.1016/j.bioorg.2020.104468.

12. El-Batal, A.I.; Al-Hazmi, N.E.; Mosallam, F.M.; El-Sayyad, G.S. Biogenic Synthesis of Copper Nanoparticles by Natural Polysaccharides and Pleurotus Ostreatus Fermented Fenugreek Using Gamma Rays with Antioxidant and Antimicrobial Potential towards Some Wound Pathogens. *Microb Pathog* **2018**, *118*, 159–169, doi:10.1016/j.micpath.2018.03.013.

13. Erci, F.; Cakir-Koc, R.; Yontem, M.; Torlak, E. Synthesis of Biologically Active Copper Oxide Nanoparticles as Promising Novel Antibacterial-Antibiofilm Agents. *Prep Biochem Biotechnol* **2020**, *50*, 538–548, doi:10.1080/10826068.2019.1711393.

14. Ghadiri, A.M.; Rabiee, N.; Bagherzadeh, M.; Kiani, M.; Fatahi, Y.; Di Bartolomeo, A.; Dinarvand, R.; Webster, T.J. Green Synthesis of CuO- And Cu2O-NPs in Assistance with High-Gravity- And Flowering of Nanobiotechnology. *Nanotechnology* **2020**, *31*, doi:10.1088/1361-6528/aba142.

15. Gu, H.; Chen, X.; Chen, F.; Zhou, X.; Parsaee, Z. Ultrasound-Assisted Biosynthesis of CuO-NPs Using Brown Alga Cystoseira Trinodis: Characterization, Photocatalytic AOP, DPPH Scavenging and Antibacterial Investigations. *Ultrason Sonochem* **2018**, *41*, 109–119, doi:10.1016/j.ultsonch.2017.09.006.

16. Hasheminya, S.-M.; Dehghannya, J. Green Synthesis and Characterization of Copper Nanoparticles Using Eryngium Caucasicum Trautv Aqueous Extracts and Its Antioxidant and Antimicrobial Properties. *Particulate Science and Technology* **2020**, *38*, 1019–1026, doi:10.1080/02726351.2019.1658664.

17. Hassan, S.E.-D.; Fouda, A.; Radwan, A.A.; Salem, S.S.; Barghoth, M.G.; Awad, M.A.; Abdo, A.M.; El-Gamal, M.S. Endophytic Actinomycetes Streptomyces Spp Mediated Biosynthesis of Copper Oxide Nanoparticles as a Promising Tool for Biotechnological Applications. *Journal of Biological Inorganic Chemistry* **2019**, doi:10.1007/s00775-019-01654-5.

18. Hayat, K.; Ali, S.; Ullah, S.; Fu, Y.; Hussain, M. Green Synthesized Silver and Copper Nanoparticles Induced Changes in Biomass Parameters, Secondary Metabolites Production, and Antioxidant Activity in Callus Cultures of Artemisia Absinthium L. *Green Processing and Synthesis* **2021**, *10*, 61–72, doi:10.1515/gps-2021-0010.

19. Ijaz, F.; Shahid, S.; Khan, S.A.; Ahmad, W.; Zaman, S. Green Synthesis of Copper Oxide Nanoparticles Using Abutilon Indicum Leaf Extract: Antimicrobial, Antioxidant and Photocatalytic Dye Degradation Activities. *Tropical Journal of Pharmaceutical Research* **2017**, *16*, 743–753, doi:10.4314/tjpr.v16i4.2.

20. Jadhav, M.S.; Kulkarni, S.; Raikar, P.; Barretto, D.A.; Vootla, S.K.; Raikar, U.S. Green Biosynthesis of CuO & Ag-CuO Nanoparticles from Malus Domestica Leaf Extract and Evaluation of Antibacterial, Antioxidant and DNA Cleavage Activities. *New Journal of Chemistry* **2018**, *42*, 204–213, doi:10.1039/c7nj02977b.

21. Jasrotia, T.; Chaudhary, S.; Kaushik, A.; Kumar, R.; Chaudhary, G.R. Green Chemistry-Assisted Synthesis of Biocompatible Ag, Cu, and Fe2O3 Nanoparticles. *Mater Today Chem* **2020**, *15*, doi:10.1016/j.mtchem.2019.100214.

22. Kalia, A.; Kaur, M.; Shami, A.; Jawandha, S.K.; Alghuthaymi, M.A.; Thakur, A.; Abd-Elsalam, K.A. Nettle-Leaf Extract Derived Zno/Cuo Nanoparticle-Biopolymer-Based Antioxidant and Antimicrobial Nanocomposite Packaging Films and Their Impact on Extending the Post-Harvest Shelf Life of Guava Fruit. *Biomolecules* **2021**, *11*, 1–24, doi:10.3390/biom11020224.

23. Lung, I.; Opriş, O.; Soran, M.-L.; Culicov, O.; Ciorîță, A.; Stegarescu, A.; Zinicovscaia, I.; Yushin, N.; Vergel, K.; Kacso, I.; et al. The Impact Assessment of Cuo Nanoparticles on the Composition and Ultrastructure of Triticum Aestivum l. *Int J Environ Res Public Health* **2021**, *18*, doi:10.3390/ijerph18136739.

24. Mahjouri, S.; Movafeghi, A.; Divband, B.; Kosari-Nasab, M. Toxicity Impacts of Chemically and Biologically Synthesized CuO Nanoparticles on Cell Suspension Cultures of Nicotiana Tabacum. *Plant Cell Tissue Organ Cult* **2018**, *135*, 223–234, doi:10.1007/s11240-018-1458-x.

25. Muthuvel, A.; Jothibas, M.; Manoharan, C. Synthesis of Copper Oxide Nanoparticles by Chemical and Biogenic Methods: Photocatalytic Degradation and in Vitro Antioxidant Activity. *Nanotechnology for Environmental Engineering* **2020**, *5*, doi:10.1007/s41204-020-00078-w.

26. Pandit, R.; Gaikwad, S.; Rai, M. Biogenic Fabrication of CuNPs, Cu Bioconjugates and in Vitro Assessment of Antimicrobial and Antioxidant Activity. *IET Nanobiotechnol* **2017**, *11*, 568–575, doi:10.1049/iet-nbt.2016.0165.

27. Rajeshkumar, S.; Menon, S.; Venkat Kumar, S.; Tambuwala, M.M.; Bakshi, H.A.; Mehta, M.; Satija, S.; Gupta, G.; Chellappan, D.K.; Thangavelu, L.; et al. Antibacterial and Antioxidant Potential of Biosynthesized Copper Nanoparticles Mediated through Cissus Arnotiana Plant Extract. *J Photochem Photobiol B* **2019**, *197*, doi:10.1016/j.jphotobiol.2019.111531.

28. Rajeshkumar, S.; Nandhini, N.T.; Manjunath, K.; Sivaperumal, P.; Krishna Prasad, G.; Alotaibi, S.S.; Roopan, S.M. Environment Friendly Synthesis Copper Oxide Nanoparticles and Its Antioxidant, Antibacterial Activities Using Seaweed (Sargassum Longifolium) Extract. *J Mol Struct* **2021**, *1242*, doi:10.1016/j.molstruc.2021.130724.

29. Rajeshkumar, S.; Rinitha, G. Nanostructural Characterization of Antimicrobial and Antioxidant Copper Nanoparticles Synthesized Using Novel Persea Americana Seeds. *OpenNano* **2018**, *3*, 18–27, doi:10.1016/j.onano.2018.03.001.

30. Rani, H.; Singh, S.P.; Yadav, T.P.; Khan, M.S.; Ansari, M.I.; Singh, A.K. In-Vitro Catalytic, Antimicrobial and Antioxidant Activities of Bioengineered Copper Quantum Dots Using Mangifera Indica (L.) Leaf Extract. *Mater Chem Phys* **2020**, *239*, doi:10.1016/j.matchemphys.2019.122052.

31. Rehana, D.; Mahendiran, D.; Kumar, R.S.; Rahiman, A.K. Evaluation of Antioxidant and Anticancer Activity of Copper Oxide Nanoparticles Synthesized Using Medicinally Important Plant Extracts. *Biomedicine and Pharmacotherapy* **2017**, *89*, 1067–1077, doi:10.1016/j.biopha.2017.02.101.

32. Sepasgozar, S.M.E.; Mohseni, S.; Feizyzadeh, B.; Morsali, A. Green Synthesis of Zinc Oxide and Copper Oxide Nanoparticles Using Achillea Nobilis Extract and Evaluating Their Antioxidant and Antibacterial Properties. *Bulletin of Materials Science* **2021**, *44*, doi:10.1007/s12034-021-02419-0.

33. Thakar, M.A.; Saurabh Jha, S.; Phasinam, K.; Manne, R.; Qureshi, Y.; Hari Babu, V. V X Ray Diffraction (XRD) Analysis and Evaluation of Antioxidant Activity of Copper Oxide Nanoparticles Synthesized from Leaf Extract of Cissus Vitiginea. *Mater Today Proc* **2021**, *51*, 319–324, doi:10.1016/j.matpr.2021.05.410.

34. Udayabhanu; Nethravathi, P.C.; Pavan Kumar, M.A.; Suresh, D.; Lingaraju, K.; Rajanaika, H.; Nagabhushana, H.; Sharma, S.C. Tinospora Cordifolia Mediated Facile Green Synthesis of Cupric Oxide Nanoparticles and Their Photocatalytic, Antioxidant and Antibacterial Properties. *Mater Sci Semicond Process* **2015**, *33*, 81–88, doi:10.1016/j.mssp.2015.01.034.

35. Velsankar, K.; Aswin Kumara, R.M.; Preethi, R.; Muthulakshmi, V.; Sudhahar, S. Green Synthesis of CuO Nanoparticles via Allium Sativum Extract and Its Characterizations on Antimicrobial, Antioxidant, Antilarvicidal Activities. *J Environ Chem Eng* **2020**, *8*, doi:10.1016/j.jece.2020.104123.

36. Velsankar, K.; Vinothini, V.; Sudhahar, S.; Kumar, M.K.; Mohandoss, S. Green Synthesis of CuO Nanoparticles via Plectranthus Amboinicus Leaves Extract with Its Characterization on Structural, Morphological, and Biological Properties. *Applied Nanoscience (Switzerland)* **2020**, *10*, 3953–3971, doi:10.1007/s13204-020-01504-w.

37. Venugopalan, R.; Pitchai, S.; Devarayan, K.; Swaminathan, V.C. Biogenic Synthesis of Copper Nanoparticles Using Borreria Hispida (Linn.) Extract and Its Antioxidant Activity. *Mater Today Proc* **2020**, *33*, 4023–4025, doi:10.1016/j.matpr.2020.06.419.

38. Wu, S.; Rajeshkumar, S.; Madasamy, M.; Mahendran, V. Green Synthesis of Copper Nanoparticles Using Cissus Vitiginea and Its Antioxidant and Antibacterial Activity against Urinary Tract Infection Pathogens. *Artif Cells Nanomed Biotechnol* **2020**, *48*, 1153–1158, doi:10.1080/21691401.2020.1817053.

39. Zangeneh, M.M.; Ghaneialvar, H.; Akbaribazm, M.; Ghanimatdan, M.; Abbasi, N.; Goorani, S.; Pirabbasi, E.; Zangeneh, A. Novel Synthesis of Falcaria Vulgaris Leaf Extract Conjugated Copper Nanoparticles with Potent Cytotoxicity, Antioxidant, Antifungal, Antibacterial, and Cutaneous Wound Healing Activities under in Vitro and in Vivo Condition. *J Photochem Photobiol B* **2019**, *197*, doi:10.1016/j.jphotobiol.2019.111556.

40. Agila, A.; Vimala, J.R.; Bharathy, M.; Dayana Jeyaleela, G.; Sheela, S.M. Anti-Oxidant and Anti-Cancer Activities of Biogenic Synthesized Copper Oxide Nanoparticles. *Biomedical and Biotechnology Research Journal (BBRJ)* **2022**, *6*, 341, doi:10.4103/bbrj.bbrj_136_22.

41. Adeyemi, J.O.; Onwudiwe, D.C.; Oyedeji, A.O. Biogenic Synthesis of CuO, ZnO, and CuO–ZnO Nanoparticles Using Leaf Extracts of Dovyalis Caffra and Their Biological Properties. *Molecules* **2022**, *27*, doi:10.3390/molecules27103206.

42. Ahamed, A.J.; Kumar, P.V.; Loganathan, K.; Karthikeyan, C.; Hameed, A.S.H. Synthesis, Characterization and Cytotoxicity Studies of CuO Nanoparticles by Using Gymnema Sylvestre Leaf Extracts. *J Indian Chem Soc* **2016**, *93*, 655–660.

43. Amin, F.; Fozia; Khattak, B.; Alotaibi, A.; Qasim, M.; Ahmad, I.; Ullah, R.; Bourhia, M.; Gul, A.; Zahoor, S.; et al. Green Synthesis of Copper Oxide Nanoparticles Using Aerva Javanica Leaf Extract and Their Characterization and Investigation of in Vitro Antimicrobial Potential and Cytotoxic Activities. *Evidence-based Complementary and Alternative Medicine* **2021**, *2021*, doi:10.1155/2021/5589703.

44. Biresaw, S.S.; Taneja, P. Copper Nanoparticles Green Synthesis and Characterization as Anticancer Potential in Breast Cancer Cells (MCF7) Derived from Prunus Nepalensis Phytochemicals. In Proceedings of the Materials Today: Proceedings; Elsevier Ltd, 2020; Vol. 49, pp. 3501–3509.

45. Chen, H.; Feng, X.; Gao, L.; Mickymaray, S.; Paramasivam, A.; Abdulaziz Alfaiz, F.; Almasmoum, H.A.; Ghaith, M.M.; Almaimani, R.A.; Aziz Ibrahim, I.A. Inhibiting the PI3K/AKT/MTOR Signalling Pathway with Copper Oxide Nanoparticles from Houttuynia Cordata Plant: Attenuating the Proliferation of Cervical Cancer Cells. *Artif Cells Nanomed Biotechnol* **2021**, *49*, 240–249, doi:10.1080/21691401.2021.1890101.

46. Dulta, K.; Ağçeli, G.K.; Chauhan, P.; Chauhan, P.K. Biogenic Production and Characterization of CuO Nanoparticles by Carica Papaya Leaves and Its Biocompatibility Applications. *J Inorg Organomet Polym Mater* **2021**, *31*, 1846–1857, doi:10.1007/s10904-020-01837-7.

47. Faisal, S.; Al-Radadi, N.S.; Jan, H.; Abdullah; Shah, S.A.; Shah, S.; Rizwan, M.; Afsheen, Z.; Hussain, Z.; Uddin, M.N.; et al. Curcuma Longa Mediated Synthesis of Copper Oxide, Nickel Oxide and Cu-Ni Bimetallic Hybrid Nanoparticles: Characterization and Evaluation for Antimicrobial, Anti-Parasitic and Cytotoxic Potentials. *Coatings* **2021**, *11*, doi:10.3390/coatings11070849.

48. Fakhar-e-Alam, M.; Shafiq, Z.; Mahmood, A.; Atif, M.; Anwar, H.; Hanif, A.; Yaqub, N.; Farooq, W.A.; Fatehmulla, A.; Ahmad, S.; et al. Assessment of Green and Chemically Synthesized Copper Oxide Nanoparticles against Hepatocellular Carcinoma. *J King Saud Univ Sci* **2021**, *33*, doi:10.1016/j.jksus.2021.101669.

49. Fouda, A.; Hassan, S.E.D.; Eid, A.M.; Awad, M.A.; Althumayri, K.; Badr, N.F.; Hamza, M.F. Endophytic Bacterial Strain, Brevibacillus Brevis-Mediated Green Synthesis of Copper Oxide Nanoparticles, Characterization, Antifungal, in Vitro Cytotoxicity, and Larvicidal Activity. *Green Processing and Synthesis* **2022**, *11*, 931–950, doi:10.1515/gps-2022-0080.

50. G, S.; S, R.; E, P.; Alhadlaq, H.A.; Mohan, R.; G, A.; Ahamed, M. Green and Chemical Synthesis of CuO Nanoparticles: A Comparative Study for Several in Vitro Bioactivities and in Vivo Toxicity in Zebrafish Embryos. *J King Saud Univ Sci* **2022**, *34*, doi:10.1016/j.jksus.2022.102092.

51. Gamedze, N.P.; Mthiyane, D.M.N.; Babalola, O.O.; Singh, M.; Onwudiwe, D.C. Physico-Chemical Characteristics and Cytotoxicity Evaluation of CuO and TiO2 Nanoparticles Biosynthesized Using Extracts of Mucuna Pruriens Utilis Seeds. *Heliyon* **2022**, *8*, doi:10.1016/j.heliyon.2022.e10187.

52. Gnanavel, V.; Palanichamy, V.; Roopan, S.M. Biosynthesis and Characterization of Copper Oxide Nanoparticles and Its Anticancer Activity on Human Colon Cancer Cell Lines (HCT-116). *J Photochem Photobiol B* **2017**, *171*, 133–138, doi:10.1016/j.jphotobiol.2017.05.001.

53. Gu, J.; Aidy, A.; Goorani, S. Anti-Human Lung Adenocarcinoma, Cytotoxicity, and Antioxidant Potentials of Copper Nanoparticles Green-Synthesized by Calendula Officinalis. *J Exp Nanosci* **2022**, *17*, 285–296, doi:10.1080/17458080.2022.2066082.

54. Hui, H.; Esmaeili, E.; Tayebee, R.; He, Q.; Abbaspour, S.; Akram, M.; Jalili, Z.; Mahdizadeh, N.; Ahmadi, A. Biosynthesis, Characterization, and Application of Cu2O Nanoparticles Originated from Cressa Leaf Extract as an Efficient Green Catalyst in the

Synthesis of Some Chromenes. *Journal of the Iranian Chemical Society* **2022**, *19*, 1261–1270, doi:10.1007/s13738-021-02378-7.

55. Emima Jeronsia, J.; Allwin Joseph, L.; Annie Vinosha, P.; Jerline Mary, A.; Jerome Das, S. Camellia Sinensis Leaf Extract Mediated Synthesis of Copper Oxide Nanostructures for Potential Biomedical Applications. *Mater Today Proc* **2019**, *8*, 214–222, doi:10.1016/j.matpr.2019.02.103.

56. Kannan, K.; Radhika, D.; Vijayalakshmi, S.; Sadasivuni, K.K.; A. Ojiaku, A.; Verma, U. Facile Fabrication of CuO Nanoparticles via Microwave-Assisted Method: Photocatalytic, Antimicrobial and Anticancer Enhancing Performance. *Int J Environ Anal Chem* **2022**, *102*, 1095–1108, doi:10.1080/03067319.2020.1733543.

57. Liu, Y.; Zeng, Z.; Jiang, O.; Li, Y.X.; Xu, Q.; Jiang, L.J.; Yu, J.; Xu, D. Green Synthesis of CuO NPs, Characterization and Their Toxicity Potential against HepG2 Cells. *Mater Res Express* **2021**, *8*, doi:10.1088/2053-1591/abd666.

58. Liu, H.; Wang, G.; Liu, J.; Nan, K.; Zhang, J.; Guo, L.; Liu, Y. Green Synthesis of Copper Nanoparticles Using Cinnamomum Zelanicum Extract and Its Applications as a Highly Efficient Antioxidant and Anti-Human Lung Carcinoma. *J Exp Nanosci* **2021**, *16*, 411–423, doi:10.1080/17458080.2021.1991577.

59. Mahmoud, N.M.R.; Mohamed, H.I.; Ahmed, S.B.; Akhtar, S. Efficient Biosynthesis of CuO Nanoparticles with Potential Cytotoxic Activity. *Chemical Papers* **2020**, *74*, 2825–2835, doi:10.1007/s11696-020-01120-6.

60. Majid, A.; Faraj, H.R. *Green Synthesis of Copper Nanoparticles Using Aqueous Extract of Yerba Mate (Llex Paraguarients St. Hill) and Its Anticancer Activity*; 2022; Vol. 18;.

61. Manasa, D.J.; Chandrashekar, K.R.; Madhu Kumar, D.J.; Niranjana, M.; Navada, K.M. Mussaenda Frondosa L. Mediated Facile Green Synthesis of Copper Oxide Nanoparticles – Characterization, Photocatalytic and Their Biological Investigations. *Arabian Journal of Chemistry* **2021**, *14*, doi:10.1016/j.arabjc.2021.103184.

62. Mohamed, R.M.; Fawzy, E.M.; Shehab, R.A.; Ali, D.M.; Salah, R.A.; Din, E.; Abd, H.M.; Fatah, E. *Green Biosynthesis, Structural Characterization and Anticancer Activity of Copper Oxide Nanoparticles from the Brown Alga Cystoseira Myrica*; 2021; Vol. 25;.

63. Mukhopadhyay, R.; Kazi, J.; Debnath, M.C. Synthesis and Characterization of Copper Nanoparticles Stabilized with Quisqualis Indica Extract: Evaluation of Its Cytotoxicity and Apoptosis in B16F10 Melanoma Cells. *Biomedicine and Pharmacotherapy* **2018**, *97*, 1373–1385, doi:10.1016/j.biopha.2017.10.167.

64. Nagajyothi, P.C.; Muthuraman, P.; Sreekanth, T.V.M.; Kim, D.H.; Shim, J. Green Synthesis: In-Vitro Anticancer Activity of Copper Oxide Nanoparticles against Human

Cervical Carcinoma Cells. *Arabian Journal of Chemistry* **2017**, *10*, 215–225, doi:10.1016/j.arabjc.2016.01.011.

65. Nakhaeepour, Z.; Mashreghi, M.; Matin, M.M.; NakhaeiPour, A.; Housaindokht, M.R. Multifunctional CuO Nanoparticles with Cytotoxic Effects on KYSE30 Esophageal Cancer Cells, Antimicrobial and Heavy Metal Sensing Activities. *Life Sci* **2019**, *234*, doi:10.1016/j.lfs.2019.116758.

66. Naz, S.; Tabassum, S.; Freitas Fernandes, N.; Mujahid, M.; Zia, M.; Carcache de Blanco, E.J. Anticancer and Antibacterial Potential of Rhus Punjabensis and CuO Nanoparticles. *Nat Prod Res* **2020**, *34*, 720–725, doi:10.1080/14786419.2018.1495633.

67. Prasad, P.R.; Kanchi, S.; Naidoo, E.B. In-Vitro Evaluation of Copper Nanoparticles Cytotoxicity on Prostate Cancer Cell Lines and Their Antioxidant, Sensing and Catalytic Activity: One-Pot Green Approach. *J Photochem Photobiol B* **2016**, *161*, 375–382, doi:10.1016/j.jphotobiol.2016.06.008.

68. Sankar, R.; Maheswari, R.; Karthik, S.; Shivashangari, K.S.; Ravikumar, V. Anticancer Activity of Ficus Religiosa Engineered Copper Oxide Nanoparticles. *Materials Science and Engineering C* **2014**, *44*, 234–239, doi:10.1016/j.msec.2014.08.030.

69. Saravanakumar, K.; Shanmugam, S.; Varukattu, N.B.; MubarakAli, D.; Kathiresan, K.; Wang, M.H. Biosynthesis and Characterization of Copper Oxide Nanoparticles from Indigenous Fungi and Its Effect of Photothermolysis on Human Lung Carcinoma. *J Photochem Photobiol B* **2019**, *190*, 103–109, doi:10.1016/j.jphotobiol.2018.11.017.

70. Shahabadi, N.; Zendehcheshm, S.; Khademi, F. Green Synthesis, in Vitro Cytotoxicity, Antioxidant Activity and Interaction Studies of CuO Nanoparticles with DNA, Serum Albumin, Hemoglobin and Lysozyme. *ChemistrySelect* **2022**, *7*, doi:10.1002/slct.202202916.

71. Shanmuga Sundaram, C.; Sivakumar, J.; Suresh Kumar, S.; Ramesh, P.; Zin, T.; Mahadeva Rao, U. *Antibacterial and Anticancer Potential of Brassica Oleracea Var Acephala Using Biosynthesised Copper Nanoparticles*;

72. Siddiquee, M.A.; Parray, M. ud din; Kamli, M.R.; Malik, M.A.; Mehdi, S.H.; Imtiyaz, K.; Rizvi, M.M.A.; Rajor, H.K.; Patel, R. Biogenic Synthesis, in-Vitro Cytotoxicity, Esterase Activity and Interaction Studies of Copper Oxide Nanoparticles with Lysozyme. *Journal of Materials Research and Technology* **2021**, *13*, 2066–2077, doi:10.1016/j.jmrt.2021.05.078.

73. Sivaraj, R.; Rahman, P.K.S.M.; Rajiv, P.; Narendhran, S.; Venckatesh, R. Biosynthesis and Characterization of Acalypha Indica Mediated Copper Oxide Nanoparticles and Evaluation of Its Antimicrobial and Anticancer Activity. *Spectrochim Acta A Mol Biomol Spectrosc* **2014**, *129*, 255–258, doi:10.1016/j.saa.2014.03.027.

74. Sonbol, H.; AlYahya, S.; Ameen, F.; Alsamhary, K.; Alwakeel, S.; Al-Otaibi, S.; Korany, S. Bioinspired Synthesize of CuO Nanoparticles Using Cylindrospermum Stagnale for Antibacterial, Anticancer and Larvicidal Applications. *Applied Nanoscience (Switzerland)* **2021**, doi:10.1007/s13204-021-01940-2.

75. Sulaiman, G.M.; Tawfeeq, A.T.; Jaaffer, M.D. Biogenic Synthesis of Copper Oxide Nanoparticles Using Olea Europaea Leaf Extract and Evaluation of Their Toxicity Activities: An in Vivo and in Vitro Study. *Biotechnol Prog* **2018**, *34*, 218–230, doi:10.1002/btpr.2568.

76. Tabrez, S.; Khan, A.U.; Mirza, A.A.; Suhail, M.; Jabir, N.R.; Zughaibi, T.A.; Alam, M. Biosynthesis of Copper Oxide Nanoparticles and Its Therapeutic Efficacy against Colon Cancer. *Nanotechnol Rev* **2022**, *11*, 1322–1331, doi:10.1515/ntrev-2022-0081.

77. Vincent, J.; Lekha, N.C. Bio-Engineered Copper Oxide Nanoparticles Using Citrus Aurantifolia Enzyme Extract and Its Anticancer Activity. *J Clust Sci* **2022**, *33*, 45–53, doi:10.1007/s10876-020-01940-2.

78. Yu, Y.; Fei, Z.; Cui, J.; Miao, B.; Lu, Y.; Wu, J. Biosynthesis of Copper Oxide Nanoparticles and Their in Vitro Cytotoxicity towards Nasopharynx Cancer (KB Cells) Cell Lines. *International Journal of Pharmacology* **2018**, *14*, 609–614, doi:10.3923/ijp.2018.609.614.

79. Yugandhar, P.; Vasavi, T.; Uma Maheswari Devi, P.; Savithramma, N. Bioinspired Green Synthesis of Copper Oxide Nanoparticles from Syzygium Alternifolium (Wt.) Walp: Characterization and Evaluation of Its Synergistic Antimicrobial and Anticancer Activity. *Applied Nanoscience (Switzerland)* **2017**, *7*, 417–427, doi:10.1007/s13204-017-0584-9.

80. Zughaibi, T.A.; Mirza, A.A.; Suhail, M.; Jabir, N.R.; Zaidi, S.K.; Wasi, S.; Zawawi, A.; Tabrez, S. Evaluation of Anticancer Potential of Biogenic Copper Oxide Nanoparticles (CuO NPs) against Breast Cancer. *J Nanomater* **2022**, *2022*, doi:10.1155/2022/5326355.

81. Zhuang, X.; Kang, Y.; Zhao, L.; Guo, S. Design and Synthesis of Copper Nanoparticles for the Treatment of Human Esophageal Cancer: Introducing a Novel Chemotherapeutic Supplement. *J Exp Nanosci* **2022**, *17*, 274–284, doi:10.1080/17458080.2022.2065264.

82. Vinu, D.; Govindaraju, K.; Vasantharaja, R.; Amreen Nisa, S.; Kannan, M.; Vijai Anand, K. Biogenic Zinc Oxide, Copper Oxide and Selenium Nanoparticles: Preparation, Characterization and Their Anti-Bacterial Activity against Vibrio Parahaemolyticus. *J Nanostructure Chem* **2021**, *11*, 271–286, doi:10.1007/s40097-020-00365-7.

83. Suresh, S.; Vennila, S.; Anita Lett, J.; Fatimah, I.; Mohammad, F.; Al-Lohedan, H.A.; Alshahateet, S.F.; Motalib Hossain, M.A.; Rafie Johan, M. Star Fruit Extract-Mediated Green Synthesis of Metal Oxide Nanoparticles. *Inorganic and Nano-Metal Chemistry* **2022**, *52*, 173–180, doi:10.1080/24701556.2021.1880437.

84. Shehabeldine, A.M.; Amin, B.H.; Hagras, F.A.; Ramadan, A.A.; Kamel, M.R.; Ahmed, M.A.; Atia, K.H.; Salem, S.S. Potential Antimicrobial and Antibiofilm Properties of Copper Oxide Nanoparticles: Time-Kill Kinetic Essay and Ultrastructure of Pathogenic Bacterial Cells. *Appl Biochem Biotechnol* **2022**, doi:10.1007/s12010-022-04120-2.

85. Sharma, S.; Kumar, K.; Thakur, N.; Chauhan, S.; Chauhan, M.S. Eco-Friendly Ocimum Tenuiflorum Green Route Synthesis of CuO Nanoparticles: Characterizations on Photocatalytic and Antibacterial Activities. *J Environ Chem Eng* **2021**, *9*, doi:10.1016/j.jece.2021.105395.

86. Sharma, S.; Kumar, K. Aloe-Vera Leaf Extract as a Green Agent for the Synthesis of CuO Nanoparticles Inactivating Bacterial Pathogens and Dye. *J Dispers Sci Technol* **2020**, 1–13, doi:10.1080/01932691.2020.1791719.

87. Sackey, J.; Razanamahandry, L.C.; Ntwampe, S.K.O.; Mlungisi, N.; Fall, A.; Kaonga, C.; Nuru, Z.Y. Biosynthesis of CuO Nanoparticles Using Mimosa Hamata Extracts. *Mater Today Proc* **2019**, *36*, 540–548, doi:10.1016/j.matpr.2020.05.325.

88. Nagore, P.; Ghotekar, S.; Mane, K.; Ghoti, A.; Bilal, M.; Roy, A. Structural Properties and Antimicrobial Activities of Polyalthia Longifolia Leaf Extract-Mediated CuO Nanoparticles. *Bionanoscience* **2021**, *11*, 579–589, doi:10.1007/s12668-021-00851-4.

89. Arya, A.; Gupta, K.; Chundawat, T.S.; Vaya, D. Biogenic Synthesis of Copper and Silver Nanoparticles Using Green Alga Botryococcus Braunii and Its Antimicrobial Activity. *Bioinorg Chem Appl* **2018**, *2018*, doi:10.1155/2018/7879403.

90. Nadeem, A.; Sumbal; Ali, J.S.; Latif, M.; Rizvi, Z.F.; Naz, S.; Mannan, A.; Zia, M. Green Synthesis and Characterization of Fe, Cu and Mg Oxide Nanoparticles Using Clematis Orientalis Leaf Extract: Salt Concentration Modulates Physiological and Biological Properties. *Mater Chem Phys* **2021**, *271*, doi:10.1016/j.matchemphys.2021.124900.

91. Ul-Hamid, A.; Dafalla, H.; Hakeem, A.S.; Haider, A.; Ikram, M. In-Vitro Catalytic and Antibacterial Potential of Green Synthesized CuO Nanoparticles against Prevalent Multiple Drug Resistant Bovine Mastitogen Staphylococcus Aureus. *Int J Mol Sci* **2022**, *23*, doi:10.3390/ijms23042335.

92. Shanmugapriya, J.; Reshma, C.A.; Srinidhi, V.; Harithpriya, K.; Ramkumar, K.M.; Umpathy, D.; Gunasekaran, K.; Subashini, R. Green Synthesis of Copper Nanoparticles Using Withania Somnifera and Its Antioxidant and Antibacterial Activity. *J Nanomater* **2022**, *2022*, doi:10.1155/2022/7967294.

93. Razavi, R.; Molaei, R.; Moradi, M.; Tajik, H.; Ezati, P.; Shafipour Yordshahi, A. Biosynthesis of Metallic Nanoparticles Using Mulberry Fruit (Morus Alba L.) Extract for the Preparation of Antimicrobial Nanocellulose Film. *Applied Nanoscience (Switzerland)* **2020**, *10*, 465–476, doi:10.1007/s13204-019-01137-8.

94. Raveesha, H.R.; Bharath, H.L.; Vasudha, D.R.; Sushma, B.K.; Pratibha, S.; Dhananjaya, N. Antibacterial and Antiproliferation Activity of Green Synthesized Nanoparticles from Rhizome Extract of Alpinia Galangal (L.) Wild. *Inorg Chem Commun* **2021**, *132*, doi:10.1016/j.inoche.2021.108854.

95. Prakash, S.; Elavarasan, N.; Venkatesan, A.; Subashini, K.; Sowndharya, M.; Sujatha, V. Green Synthesis of Copper Oxide Nanoparticles and Its Effective Applications in Biginelli Reaction, BTB Photodegradation and Antibacterial Activity. *Advanced Powder Technology* **2018**, *29*, 3315–3326, doi:10.1016/j.apt.2018.09.009.

96. Naseer, M.; Ramadan, R.; Xing, J.; Samak, N.A. Facile Green Synthesis of Copper Oxide Nanoparticles for the Eradication of Multidrug Resistant Klebsiella Pneumonia and Helicobacter Pylori Biofilms. *Int Biodeterior Biodegradation* **2021**, *159*, doi:10.1016/j.ibiod.2021.105201.

97. Naika, H.R.; Lingaraju, K.; Manjunath, K.; Kumar, D.; Nagaraju, G.; Suresh, D.; Nagabhushana, H. Green Synthesis of CuO Nanoparticles Using Gloriosa Superba L. Extract and Their Antibacterial Activity. *Journal of Taibah University for Science* **2015**, *9*, 7–12, doi:10.1016/j.jtusci.2014.04.006.

98. Lingaraju, K.; Raja Naika, H.; Manjunath, K.; Nagaraju, G.; Suresh, D.; Nagabhushana, H. Rauvolfia Serpentina-Mediated Green Synthesis of CuO Nanoparticles and Its Multidisciplinary Studies. *Acta Metallurgica Sinica (English Letters)* **2015**, *28*, 1134–1140, doi:10.1007/s40195-015-0304-y.

99. Kumar, P.P.N.V.; Shameem, U.; Kollu, P.; Kalyani, R.L.; Pammi, S.V.N. Green Synthesis of Copper Oxide Nanoparticles Using Aloe Vera Leaf Extract and Its Antibacterial Activity Against Fish Bacterial Pathogens. *Bionanoscience* **2015**, *5*, 135–139, doi:10.1007/s12668-015-0171-z.

100. Kalaiyan, G.; Suresh, S.; Thambidurai, S.; Prabu, K.M.; Kumar, S.K.; Pugazhenthiran, N.; Kandasamy, M. Green Synthesis of Hierarchical Copper Oxide Microleaf Bundles Using Hibiscus Cannabinus Leaf Extract for Antibacterial Application. *J Mol Struct* **2020**, *1217*, doi:10.1016/j.molstruc.2020.128379.

101. K., V.; S., S.; P., M.; S., M.; S., S. Ecofriendly Green Synthesis, Characterization and Biomedical Applications of CuO Nanoparticles Synthesized Using Leaf Extract of Capsicum Frutescens. *J Environ Chem Eng* **2021**, *9*, doi:10.1016/j.jece.2021.106299.

102. Aziz, W.J.; Abid, M.A.; Hussein, E.H. Biosynthesis of CuO Nanoparticles and Synergistic Antibacterial Activity Using Mint Leaf Extract. *Materials Technology* **2020**, *35*, 447–451, doi:10.1080/10667857.2019.1692163.

103. Angeline Mary, A.P.; Thaminum Ansari, A.; Subramanian, R. Sugarcane Juice Mediated Synthesis of Copper Oxide Nanoparticles, Characterization and Their Antibacterial Activity. *J King Saud Univ Sci* **2019**, *31*, 1103–1114, doi:10.1016/j.jksus.2019.03.003.

104. Andualem, W.W.; Sabir, F.K.; Mohammed, E.T.; Belay, H.H.; Gonfa, B.A. Synthesis of Copper Oxide Nanoparticles Using Plant Leaf Extract of Catha Edulis and Its Antibacterial Activity. *J Nanotechnol* **2020**, *2020*, doi:10.1155/2020/2932434.

105. Alishah, H.; Pourseyedi, S.; Ebrahimipour, S.Y.; Mahani, S.E.; Rafiei, N. Green Synthesis of Starch-Mediated CuO Nanoparticles: Preparation, Characterization, Antimicrobial Activities and in Vitro MTT Assay against MCF-7 Cell Line. *Rendiconti Lincei* **2017**, *28*, 65–71, doi:10.1007/s12210-016-0574-y.

106. Das, P.; Ghosh, S.; Ghosh, R.; Dam, S.; Baskey, M. Madhuca Longifolia Plant Mediated Green Synthesis of Cupric Oxide Nanoparticles: A Promising Environmentally Sustainable Material for Waste Water Treatment and Efficient Antibacterial Agent. *J Photochem Photobiol B* **2018**, *189*, 66–73, doi:10.1016/j.jphotobiol.2018.09.023.

107. Bocarando-Chacón, J.; Vargas-Vazquez, D.; Martinez-Suarez, F.; Flores-Juárez, C.; Cortez-Valadez, M. Surface-Enhanced Raman Scattering and Antibacterial Properties from Copper Nanoparticles Obtained by Green Chemistry. *Appl Phys A Mater Sci Process* **2020**, *126*, doi:10.1007/s00339-020-03704-1.

108. Vasantharaj, S.; Shivakumar, P.; Sathiyavimal, S.; Senthilkumar, P.; Vijayaram, S.; Shanmugavel, M.; Pugazhendhi, A. Antibacterial Activity and Photocatalytic Dye Degradation of Copper Oxide Nanoparticles (CuONPs) Using Justicia Gendarussa. *Applied Nanoscience (Switzerland)* **2021**, doi:10.1007/s13204-021-01939-9.

109. Vasantharaj, S.; Sathiyavimal, S.; Saravanan, M.; Senthilkumar, P.; Gnanasekaran, K.; Shanmugavel, M.; Manikandan, E.; Pugazhendhi, A. Synthesis of Ecofriendly Copper Oxide Nanoparticles for Fabrication over Textile Fabrics: Characterization of Antibacterial Activity and Dye Degradation Potential. *J Photochem Photobiol B* **2019**, *191*, 143–149, doi:10.1016/j.jphotobiol.2018.12.026.

110. Vaidehi, D.; Bhuvaneshwari, V.; Bharathi, D.; Sheetal, B.P. Antibacterial and Photocatalytic Activity of Copper Oxide Nanoparticles Synthesized Using Solanum Lycopersicum Leaf Extract. *Mater Res Express* **2018**, *5*, doi:10.1088/2053-1591/aad426.

111. Tamil Elakkiya, V.; Meenakshi, R. V; Senthil Kumar, P.; Karthik, V.; Ravi Shankar, K.; Sureshkumar, P.; Hanan, A. Green Synthesis of Copper Nanoparticles Using Sesbania Aculeata to Enhance the Plant Growth and Antimicrobial Activities. *International Journal of Environmental Science and Technology* **2022**, *19*, 1313–1322, doi:10.1007/s13762-021-03182-9.

112. Tahvilian, R.; Zangeneh, M.M.; Falahi, H.; Sadrjavadi, K.; Jalalvand, A.R.; Zangeneh, A. Green Synthesis and Chemical Characterization of Copper Nanoparticles Using Allium Saralicum Leaves and Assessment of Their Cytotoxicity, Antioxidant, Antimicrobial, and Cutaneous Wound Healing Properties. *Appl Organomet Chem* **2019**, *33*, doi:10.1002/aoc.5234.

113. Taherzadeh Soureshjani, P.; Shadi, A.; Mohammadsaleh, F. Algae-Mediated Route to Biogenic Cuprous Oxide Nanoparticles and Spindle-like CaCO3: A Comparative Study, Facile Synthesis, and Biological Properties. *RSC Adv* **2021**, *11*, 10599–10609, doi:10.1039/d1ra00187f.

114. Sivaraj, R.; Rahman, P.K.S.M.; Rajiv, P.; Salam, H.A.; Venckatesh, R. Biogenic Copper Oxide Nanoparticles Synthesis Using Tabernaemontana Divaricate Leaf Extract and Its Antibacterial Activity against Urinary Tract Pathogen. *Spectrochim Acta A Mol Biomol Spectrosc* **2014**, *133*, 178–181, doi:10.1016/j.saa.2014.05.048.

115. Shende, S.; Ingle, A.P.; Gade, A.; Rai, M. Green Synthesis of Copper Nanoparticles by Citrus Medica Linn. (Idilimbu) Juice and Its Antimicrobial Activity. *World J Microbiol Biotechnol* **2015**, *31*, 865–873, doi:10.1007/s11274-015-1840-3.

116. Seetha, J.; Mallavarapu, U.; Mesa, A. In Situ Green Synthesis of Antibacterial Copper Nanocomposite Cotton Fabrics Using Achyranthes Aspera Leaf Extract. *J Appl Pharm Sci* **2020**, *10*, 104–109, doi:10.7324/JAPS.2020.10514.

117. Roy, K.; Sarkar, C.K.; Ghosh, C.K. Antibacterial Mechanism of Biogenic Copper Nanoparticles Synthesized Using Heliconia Psittacorum Leaf Extract. *Nanotechnol Rev* **2016**, *5*, 529–536, doi:10.1515/ntrev-2016-0040.

118. Ramzan, M.; Obodo, R.M.; Mukhtar, S.; Ilyas, S.Z.; Aziz, F.; Thovhogi, N. Green Synthesis of Copper Oxide Nanoparticles Using Cedrus Deodara Aqueous Extract for Antibacterial Activity. *Mater Today Proc* **2019**, *36*, 576–581, doi:10.1016/j.matpr.2020.05.472.

119. Rafique, M.; Tahir, M.B.; Irshad, M.; Nabi, G.; Gillani, S.S.A.; Iqbal, T.; Mubeen, M. Novel Citrus Aurantifolia Leaves Based Biosynthesis of Copper Oxide Nanoparticles for Environmental and Wastewater Purification as an Efficient Photocatalyst and Antibacterial Agent. *Optik (Stuttg)* **2020**, *219*, doi:10.1016/j.ijleo.2020.165138.

120. Nagaraj, E.; Karuppannan, K.; Shanmugam, P.; Venugopal, S. Exploration of Bio-Synthesized Copper Oxide Nanoparticles Using Pterolobium Hexapetalum Leaf Extract by Photocatalytic Activity and Biological Evaluations. *J Clust Sci* **2019**, *30*, 1157–1168, doi:10.1007/s10876-019-01579-8.

121. Hosseinzadeh, R.; Mohadjerani, M.; Mesgar, S. Green Synthesis of Copper Oxide Nanoparticles Using Aqueous Extract of Convolvulus Percicus L. as Reusable Catalysts

in Cross-Coupling Reactions and Their Antibacterial Activity. *IET Nanobiotechnol* **2017**, *11*, 725–730, doi:10.1049/iet-nbt.2016.0241.

122. Gopalakrishnan, V.; Muniraj, S. Neem Flower Extract Assisted Green Synthesis of Copper Nanoparticles - Optimisation, Characterisation and Anti-Bacterial Study. *Mater Today Proc* **2019**, *36*, 832–836, doi:10.1016/j.matpr.2020.07.013.

123. Gholami, M.; Azarbani, F.; Hadi, F.; Murthy, H.C.A. Eco-Friendly Synthesis of Copper Nanoparticles Using Mentha Pulegium Leaf Extract: Characterisation, Antibacterial and Cytotoxic Activities. *Materials Technology* **2022**, *37*, 1523–1531, doi:10.1080/10667857.2021.1959214.

124. Fernandez, A.C.; KM, A.; Rajagopal, R. Green Synthesis, Characterization, Catalytic and Antibacterial Studies of Copper Iodide Nanoparticles Synthesized Using Brassica Oleracea Var. Capitata f. Rubra Extract. *Chemical Data Collections* **2020**, *29*, doi:10.1016/j.cdc.2020.100538.

125. Fatma, S.; Kalainila, P.; Fatma, S.; Renganathan, S. Green Synthesis of Copper Nanoparticle from Passiflora Foetida Leaf Extract and Its Antibacterial Activity. *Asian Journal of Pharmaceutical and Clinical Research* **2017**, *10*, 79–83, doi:10.22159/ajpcr.2017.v10i4.15744.

126. Zaman, M.B.; Poolla, R.; Singh, P.; Gudipati, T. Biogenic Synthesis of CuO Nanoparticles Using Tamarindus Indica L. and a Study of Their Photocatalytic and Antibacterial Activity. *Environ Nanotechnol Monit Manag* **2020**, *14*, doi:10.1016/j.enmm.2020.100346.

127. Velmurugan, P.; Jang, S.H.; Hong, S.C.; Yi, P.I.; Jung, E.S.; Park, J.S.; Sivakumar, S. Green Crystallization and Characterization of Copper Oxide (CuO) Nanoparticles Using Anacardium Occidentale Shell Liquid and Their Biomedical Applications. *Journal of Nano Research* **2016**, *40*, 167–173, doi:10.4028/www.scientific.net/JNanoR.40.167.

128. Taha, J.H.; Abbas, N.K.; Al-Attraqchi, A.A.F. Green Synthesis and Evaluation of Copper Oxide Nanoparticles Using Fig Leaves and Their Antifungal and Antibacterial Activities. *International Journal of Drug Delivery Technology* **2020**, *10*, 378–382, doi:10.25258/ijddt.10.3.13.

129. Sharma, P.; Pant, S.; Dave, V.; Tak, K.; Sadhu, V.; Reddy, K.R. Green Synthesis and Characterization of Copper Nanoparticles by Tinospora Cardifolia to Produce Nature-Friendly Copper Nano-Coated Fabric and Their Antimicrobial Evaluation. *J Microbiol Methods* **2019**, *160*, 107–116, doi:10.1016/j.mimet.2019.03.007.

130. Reddeppa, M.; Reddy, R.C.K.; Raj, Y.P.; Rani, T.S. Green Synthesis of Copper Nanoparticles: Evaluation of Catalytic and Antibacterial Activity. *Asian Journal of Chemistry* **2019**, *31*, 622–626, doi:10.14233/ajchem.2019.21750.

131. Jain, V.; Khusnud, A.; Tiwari, J.; Mishra, M.; Mishra, P.K. Biogenic Proceedings and Characterization of Copper-Gold Nanoalloy: Evaluation of Their Innate Antimicrobial and Catalytic Activities. *Inorganic and Nano-Metal Chemistry* **2021**, *51*, 230–238, doi:10.1080/24701556.2020.1783313.

132. Fazal, A.; Ara, S.; Ishaq, M.T.; Sughra, K. Green Fabrication of Copper Oxide Nanoparticles: A Comparative Antibacterial Study Against Gram-Positive and Gram-Negative Bacteria. *Arab J Sci Eng* **2022**, *47*, 523–533, doi:10.1007/s13369-021-05767-5.

133. Benassai, E.; Del Bubba, M.; Ancillotti, C.; Colzi, I.; Gonnelli, C.; Calisi, N.; Salvatici, M.C.; Casalone, E.; Ristori, S. Green and Cost-Effective Synthesis of Copper Nanoparticles by Extracts of Non-Edible and Waste Plant Materials from Vaccinium Species: Characterization and Antimicrobial Activity. *Materials Science and Engineering C* **2021**, *119*, doi:10.1016/j.msec.2020.111453.

134. Manjari, G.; Saran, S.; Arun, T.; Vijaya Bhaskara Rao, A.; Devipriya, S.P. Catalytic and Recyclability Properties of Phytogenic Copper Oxide Nanoparticles Derived from Aglaia Elaeagnoidea Flower Extract. *Journal of Saudi Chemical Society* **2017**, *21*, 610–618, doi:10.1016/j.jscs.2017.02.004.

135. Nasrollahzadeh, M.; Momeni, S.S.; Sajadi, S.M. Green Synthesis of Copper Nanoparticles Using Plantago Asiatica Leaf Extract and Their Application for the Cyanation of Aldehydes Using K 4 Fe(CN) 6. *J Colloid Interface Sci* **2017**, *506*, 471–477, doi:10.1016/j.jcis.2017.07.072.

136. Devi, T.B.; Ahmaruzzaman, M. Removal of Perilous Nitrocompound from Aqueous Phase Using Biogenic Copper Nanoparticles as a Catalyst. *Indian Journal of Chemical Technology* **2018**, *25*, 561–564.

137. Bagherzadeh, M.; Safarkhani, M.; Ghadiri, A.M.; Kiani, M.; Fatahi, Y.; Taghavimandi, F.; Daneshgar, H.; Abbariki, N.; Makvandi, P.; Varma, R.S.; et al. Bioengineering of CuO Porous (Nano)Particles: Role of Surface Amination in Biological, Antibacterial, and Photocatalytic Activity. *Sci Rep* **2022**, *12*, doi:10.1038/s41598-022-19553-2.

138. Kiriyanthan, R.M.; Sharmili, S.A.; Balaji, R.; Jayashree, S.; Mahboob, S.; Al-Ghanim, K.A.; Al-Misned, F.; Ahmed, Z.; Govindarajan, M.; Vaseeharan, B. Photocatalytic, Antiproliferative and Antimicrobial Properties of Copper Nanoparticles Synthesized Using Manilkara Zapota Leaf Extract: A Photodynamic Approach. *Photodiagnosis Photodyn Ther* **2020**, *32*, doi:10.1016/j.pdpdt.2020.102058.

139. Veisi, H.; Karmakar, B.; Tamoradi, T.; Hemmati, S.; Hekmati, M.; Hamelian, M. Biosynthesis of CuO Nanoparticles Using Aqueous Extract of Herbal Tea (Stachys Lavandulifolia) Flowers and Evaluation of Its Catalytic Activity. *Sci Rep* **2021**, *11*, doi:10.1038/s41598-021-81320-6.

140. Kushwah, M.; Yadav, R.; Gaur, M.S.; Berlina, A.N. Copper Nanoparticles-Catalysed Reduction of Methylene Blue and High-Sensitive Chemiluminescence Detection of Mercury. *Int J Environ Anal Chem* **2021**, doi:10.1080/03067319.2021.1893706.

141. Siddiqi, K.S.; Rashid, M.; Rahman, A.; Tajuddin; Husen, A.; Rehman, S. Green Synthesis, Characterization, Antibacterial and Photocatalytic Activity of Black Cupric Oxide Nanoparticles. *Agric Food Secur* **2020**, *9*, doi:10.1186/s40066-020-00271-9.

142. Singh, J.; Kumar, V.; Kim, K.-H.; Rawat, M. Biogenic Synthesis of Copper Oxide Nanoparticles Using Plant Extract and Its Prodigious Potential for Photocatalytic Degradation of Dyes. *Environ Res* **2019**, *177*, doi:10.1016/j.envres.2019.108569.

143. Kerour, A.; Boudjadar, S.; Bourzami, R.; Allouche, B. Eco-Friendly Synthesis of Cuprous Oxide (Cu2O) Nanoparticles and Improvement of Their Solar Photocatalytic Activities. *J Solid State Chem* **2018**, *263*, 79–83, doi:10.1016/j.jssc.2018.04.010.

144. Phang, Y.-K.; Aminuzzaman, M.; Akhtaruzzaman, M.; Muhammad, G.; Ogawa, S.; Watanabe, A.; Tey, L.-H. Green Synthesis and Characterization of CuO Nanoparticles Derived from Papaya Peel Extract for the Photocatalytic Degradation of Palm Oil Mill Effluent (POME). *Sustainability (Switzerland)* **2021**, *13*, 1–15, doi:10.3390/su13020796.

145. Devi, T.B.; Ahmaruzzaman, M. Facile Preparation of Copper Nanaoparticles Using Coccinia Grandis Fruit Extract and Its Application towards the Reduction of Toxic Nitro Compound. *Mater Today Proc* **2018**, *5*, 2098–2104, doi:10.1016/j.matpr.2017.09.206.

146. Kumar, B.; Smita, K.; Debut, A.; Cumbal, L. Andean Sacha Inchi (Plukenetiavolubilis l.) Leaf-Mediated Synthesis of Cu2O Nanoparticles: A Low-Cost Approach. *Bioengineering* **2020**, *7*, 1–10, doi:10.3390/bioengineering7020054.

147. Nazar, N.; Bibi, I.; Kamal, S.; Iqbal, M.; Nouren, S.; Jilani, K.; Umair, M.; Ata, S. Cu Nanoparticles Synthesis Using Biological Molecule of P. Granatum Seeds Extract as Reducing and Capping Agent: Growth Mechanism and Photo-Catalytic Activity. *Int J Biol Macromol* **2018**, *106*, 1203–1210, doi:10.1016/j.ijbiomac.2017.08.126.

148. Prabhu, S.; Thangadurai, T.D.; Bharathy, P. V; Kalugasalam, P. Investigation on the Photocatalytic and Antibacterial Activities of Green Synthesized Cupric Oxide Nanoparticles Using Clitoria Ternatea. *Iranian Journal of Catalysis* **2022**, *12*, doi:10.30495/ijc.2022.689547.

149. Subha, V.; Kirubanandan, S.; Arulmozhi, M.; Renganathan, S. Green Synthesis of Copper Nanoparticles Using o Dina Woider Gum Extract and Their Effect on Photocatalytic Dye Degradation. *Chemist* **2018**, *91*, 9–19.

150. Veisi, H.; Hemmati, S.; Javaheri, H. N-Arylation of Indole and Aniline by a Green Synthesized CuO Nanoparticles Mediated by Thymbra Spicata Leaves Extract as a

Recyclable and Heterogeneous Nanocatalyst. *Tetrahedron Lett* **2017**, *58*, 3155–3159, doi:10.1016/j.tetlet.2017.06.086.

151. Ssekatawa, K.; Byarugaba, D.K.; Angwe, M.K.; Wampande, E.M.; Ejobi, F.; Nxumalo, E.; Maaza, M.; Sackey, J.; Kirabira, J.B. Phyto-Mediated Copper Oxide Nanoparticles for Antibacterial, Antioxidant and Photocatalytic Performances. *Front Bioeng Biotechnol* **2022**, *10*, doi:10.3389/fbioe.2022.820218.

152. Chowdhury, R.; Khan, A.; Rashid, M.H. Green Synthesis of CuO Nanoparticles Using: Lantana Camara Flower Extract and Their Potential Catalytic Activity towards the Aza-Michael Reaction. *RSC Adv* **2020**, *10*, 14374–14385, doi:10.1039/d0ra01479f.

153. Priya, D.D.; Roopan, S.M.; Singh, S.; Bansal, J.; Shanavas, S.; Khan, M.R.; Al-Dhabi, N.A.; Arasu, M. V; Duraipandiyan, V. Phyto-Synthesis of CuO Nano-Particles and Its Catalytic Application in C-S Bond Formation. *Mater Lett* **2020**, *266*, doi:10.1016/j.matlet.2020.127486.

154. Karuppannan, S.K.; Ramalingam, R.; Mohamed Khalith, S.B.; Dowlath, M.J.H.; Darul Raiyaan, G.I.; Arunachalam, K.D. Characterization, Antibacterial and Photocatalytic Evaluation of Green Synthesized Copper Oxide Nanoparticles. *Biocatal Agric Biotechnol* **2021**, *31*, doi:10.1016/j.bcab.2020.101904.

155. Yasin, A.; Fatima, U.; Shahid, S.; Mansoor, S.; Inam, H.; Javed, M.; Iqbal, S.; Alrbyawi, H.; Somaily, H.H.; Pashameah, R.A.; et al. Fabrication of Copper Oxide Nanoparticles Using Passiflora Edulis Extract for the Estimation of Antioxidant Potential and Photocatalytic Methylene Blue Dye Degradation. *Agronomy* **2022**, *12*, doi:10.3390/agronomy12102315.

156. Ullah, H.; Ullah, Z.; Fazal, A.; Irfan, M. Use of Vegetable Waste Extracts for Controlling Microstructure of CuO Nanoparticles: Green Synthesis, Characterization, and Photocatalytic Applications. *J Chem* **2017**, *2017*, doi:10.1155/2017/2721798.

157. Olajire, A.A.; Ifediora, N.F.; Bello, M.D.; Benson, N.U. Green Synthesis of Copper Nanoparticles Using Alchornea Laxiflora Leaf Extract and Their Catalytic Application for Oxidative Desulphurization of Model Oil. *Iran J Sci Technol Trans A Sci* **2018**, *42*, 1935–1946, doi:10.1007/s40995-017-0404-9.

158. Haseena, S.; Shanavas, S.; Duraimurugan, J.; Ahamad, T.; Alshehri, S.M.; Acevedo, R.; Jayamani, N. Investigation on Photocatalytic and Antibacterial Ability of Green Treated Copper Oxide Nanoparticles Using Artabotrys Hexapetalus and Bambusa Vulgaris Plant Extract. *Mater Res Express* **2019**, *6*, doi:10.1088/2053-1591/ab59a9.

159. Al-Jubouri, A.K.; Al-Saadi, N.H.; Kadhim, M.A. Green Synthesis of Copper Nanoparticles from Myrtus Communis Leaves Extract: Characterization, Antioxidant and Catalytic Activity. *Iraqi Journal of Agricultural Sciences* **2022**, *53*, 471–486, doi:10.36103/ijas.v53i2.1555.

160. Buazar, F.; Sweidi, S.; Badri, M.; Kroushawi, F. Biofabrication of Highly Pure Copper Oxide Nanoparticles Using Wheat Seed Extract and Their Catalytic Activity: A Mechanistic Approach. *Green Processing and Synthesis* **2019**, *8*, 691–702, doi:10.1515/gps-2019-0040.

161. Ghosh, M.K.; Sahu, S.; Gupta, I.; Ghorai, T.K. Green Synthesis of Copper Nanoparticles from an Extract OfJatropha Curcasleaves: Characterization, Optical Properties, CT-DNA Binding and Photocatalytic Activity. *RSC Adv* **2020**, *10*, 22027–22035, doi:10.1039/d0ra03186k.

162. Rafique, M.; Shafiq, F.; Ali Gillani, S.S.; Shakil, M.; Tahir, M.B.; Sadaf, I. Eco-Friendly Green and Biosynthesis of Copper Oxide Nanoparticles Using Citrofortunella Microcarpa Leaves Extract for Efficient Photocatalytic Degradation of Rhodamin B Dye Form Textile Wastewater. *Optik (Stuttg)* **2020**, *208*, doi:10.1016/j.ijleo.2019.164053.

163. Alinezhad, H.; Pakzad, K. C-S Cross-Coupling Reaction Using Novel and Green Synthesized CuO Nanoparticles Assisted by Euphorbia Maculata Extract. *Appl Organomet Chem* **2019**, *33*, doi:10.1002/aoc.5144.

164. Selvam, K.; Sudhakar, C.; Selvankumar, T.; Senthilkumar, B.; Selva Kumar, R.; Kannan, N. Biomimetic Synthesis of Copper Nanoparticles Using Rhizome Extract of Corallocarbus Epigaeus and Their Bactericidal with Photocatalytic Activity. *SN Appl Sci* **2020**, *2*, doi:10.1007/s42452-020-2811-3.

165. Kayalvizhi, S.; Sengottaiyan, A.; Selvankumar, T.; Senthilkumar, B.; Sudhakar, C.; Selvam, K. Eco-Friendly Cost-Effective Approach for Synthesis of Copper Oxide Nanoparticles for Enhanced Photocatalytic Performance. *Optik (Stuttg)* **2020**, *202*, doi:10.1016/j.ijleo.2019.163507.

166. Roselyn Maheo, A.; Scholastica Mary Vithiya, B.; Augustine Arul Prasad, T.; Tamizhdurai, P.; Mangesh, V.L. Biosynthesis, Characterization, Biological and Photo Catalytic Investigations of Elsholtzia Blanda and Chitosan Mediated Copper Oxide Nanoparticles: Biosynthesis, Characterization, Biological and Photo Catalytic Investigations. *Arabian Journal of Chemistry* **2022**, *15*, doi:10.1016/j.arabjc.2021.103661.

167. Chandraker, S.K.; Lal, M.; Ghosh, M.K.; Tiwari, V.; Ghorai, T.K.; Shukla, R. Green Synthesis of Copper Nanoparticles Using Leaf Extract of Ageratum Houstonianum Mill. and Study of Their Photocatalytic and Antibacterial Activities. *Nano Express* **2020**, *1*, doi:10.1088/2632-959X/ab8e99.

168. Dulta, K.; Koşarsoy Ağçeli, G.; Chauhan, P.; Jasrotia, R.; Chauhan, P.K.; Ighalo, J.O. Multifunctional CuO Nanoparticles with Enhanced Photocatalytic Dye Degradation and Antibacterial Activity. *Sustainable Environment Research* **2022**, *32*, doi:10.1186/s42834-021-00111-w.

169. Surendhiran, S.; Gowthambabu, V.; Balamurugan, A.; Sudha, M.; Senthil Kumar, V.B.; Suresh, K.C. Rapid Green Synthesis of CuO Nanoparticles and Evaluation of Its Photocatalytic and Electrochemical Corrosion Inhibition Performance. *Mater Today Proc* **2021**, *47*, 1011–1016, doi:10.1016/j.matpr.2021.05.515.

170. Nasrollahzadeh, M.; Sajadi, S.M.; Mirzaei, Y. An Efficient One-Pot Synthesis of 1,4-Disubstituted 1,2,3-Triazoles at Room Temperature by Green Synthesized Cu NPs Using Otostegia Persica Leaf Extract. *J Colloid Interface Sci* **2016**, *468*, 156–162, doi:10.1016/j.jcis.2016.01.050.

171. Tamuly, C.; Saikia, I.; Hazarika, M.; Das, M.R. Reduction of Aromatic Nitro Compounds Catalyzed by Biogenic CuO Nanoparticles. *RSC Adv* **2014**, *4*, 53229–53236, doi:10.1039/c4ra10397a.

172. Nasrollahzadeh, M.; Sajadi, S.M.; Maham, M. Tamarix Gallica Leaf Extract Mediated Novel Route for Green Synthesis of CuO Nanoparticles and Their Application for N-Arylation of Nitrogen-Containing Heterocycles under Ligand-Free Conditions. *RSC Adv* **2015**, *5*, 40628–40635, doi:10.1039/c5ra04012d.

173. Nasrollahzadeh, M.; Mohammad Sajadi, S. Green Synthesis of Copper Nanoparticles Using Ginkgo Biloba L. Leaf Extract and Their Catalytic Activity for the Huisgen [3+2] Cycloaddition of Azides and Alkynes at Room Temperature. *J Colloid Interface Sci* **2015**, *457*, 141–147, doi:10.1016/j.jcis.2015.07.004.

174. Nasrollahzadeh, M.; Mohammad Sajadi, S.; Rostami-Vartooni, A. Green Synthesis of CuO Nanoparticles by Aqueous Extract of Anthemis Nobilis Flowers and Their Catalytic Activity for the A3 Coupling Reaction. *J Colloid Interface Sci* **2015**, *459*, 183–188, doi:10.1016/j.jcis.2015.08.020.

175. Nasrollahzadeh, M.; Sajadi, S.M.; Rostami-Vartooni, A.; Hussin, S.M. Green Synthesis of CuO Nanoparticles Using Aqueous Extract of Thymus Vulgaris L. Leaves and Their Catalytic Performance for N-Arylation of Indoles and Amines. *J Colloid Interface Sci* **2016**, *466*, 113–119, doi:10.1016/j.jcis.2015.12.018.

176. Borah, R.; Saikia, E.; Bora, S.J.; Chetia, B. On-Water Synthesis of Phenols Using Biogenic Cu2O Nanoparticles without Using H2O2. *RSC Adv* **2016**, *6*, 100443–100447, doi:10.1039/c6ra22972g.

177. Nasrollahzadeh, M.; Sajadi, S.M.; Khalaj, M. Green Synthesis of Copper Nanoparticles Using Aqueous Extract of the Leaves of Euphorbia Esula L and Their Catalytic Activity for Ligand-Free Ullmann-Coupling Reaction and Reduction of 4-Nitrophenol. *RSC Adv* **2014**, *4*, 47313–47318, doi:10.1039/c4ra08863h.

178. Nasrollahzadeh, M.; Maham, M.; Mohammad Sajadi, S. Green Synthesis of CuO Nanoparticles by Aqueous Extract of Gundelia Tournefortii and Evaluation of Their

Catalytic Activity for the Synthesis of N-Monosubstituted Ureas and Reduction of 4-Nitrophenol. *J Colloid Interface Sci* **2015**, *455*, 245–253, doi:10.1016/j.jcis.2015.05.045.

179. Sinha, T.; Ahmaruzzaman, M. Biogenic Synthesis of Cu Nanoparticles and Its Degradation Behavior for Methyl Red. *Mater Lett* **2015**, *159*, 168–171, doi:10.1016/j.matlet.2015.06.099.

180. Raina, S.; Roy, A.; Bharadvaja, N. Degradation of Dyes Using Biologically Synthesized Silver and Copper Nanoparticles. *Environ Nanotechnol Monit Manag* **2020**, *13*, 100278, doi:10.1016/J.ENMM.2019.100278.

181. Sorbiun, M.; Shayegan Mehr, E.; Ramazani, A.; Taghavi Fardood, S. Green Synthesis of Zinc Oxide and Copper Oxide Nanoparticles Using Aqueous Extract of Oak Fruit Hull (Jaft) and Comparing Their Photocatalytic Degradation of Basic Violet 3. *Int J Environ Res* **2018**, *12*, 29–37, doi:10.1007/s41742-018-0064-4.

182. Wang, G.; Zhao, K.; Gao, C.; Wang, J.; Mei, Y.; Zheng, X.; Zhu, P. Green Synthesis of Copper Nanoparticles Using Green Coffee Bean and Their Applications for Efficient Reduction of Organic Dyes. *J Environ Chem Eng* **2021**, *9*, doi:10.1016/j.jece.2021.105331.

183. Ismail, M.; Gul, S.; Khan, M.I.; Khan, M.A.; Asiri, A.M.; Khan, S.B. Green Synthesis of Zerovalent Copper Nanoparticles for Efficient Reduction of Toxic Azo Dyes Congo Red and Methyl Orange. *Green Processing and Synthesis* **2019**, *8*, 135–143, doi:10.1515/gps-2018-0038.

184. Batool, M.; Qureshi, M.Z.; Hashmi, F.; Mehboob, N.; Daoush, W.M. Adsorption of Congo Red (Acid Red 28) Azodye on Biosynthesized Copper Oxide Nanoparticles. *Asian Journal of Chemistry* **2019**, *31*, 707–713, doi:10.14233/ajchem.2019.21752.

185. Roy, K.; Ghosh, C.K.; Sarkar, C.K. Degradation of Toxic Textile Dyes and Detection of Hazardous Hg2+ by Low-Cost Bioengineered Copper Nanoparticles Synthesized Using Impatiens Balsamina Leaf Extract. *Mater Res Bull* **2017**, *94*, 257–262, doi:10.1016/j.materresbull.2017.06.016.

186. Noman, M.; Shahid, M.; Ahmed, T.; Niazi, M.B.K.; Hussain, S.; Song, F.; Manzoor, I. Use of Biogenic Copper Nanoparticles Synthesized from a Native Escherichia Sp. as Photocatalysts for Azo Dye Degradation and Treatment of Textile Effluents. *Environmental Pollution* **2020**, *257*, doi:10.1016/j.envpol.2019.113514.

187. Khani, R.; Roostaei, B.; Bagherzade, G.; Moudi, M. Green Synthesis of Copper Nanoparticles by Fruit Extract of Ziziphus Spina-Christi (L.) Willd.: Application for Adsorption of Triphenylmethane Dye and Antibacterial Assay. *J Mol Liq* **2018**, *255*, 541–549, doi:10.1016/j.molliq.2018.02.010.

188. Mahmoud, A.E.D.; Al-Qahtani, K.M.; Alflaij, S.O.; Al-Qahtani, S.F.; Alsamhan, F.A. Green Copper Oxide Nanoparticles for Lead, Nickel, and Cadmium Removal from Contaminated Water. *Sci Rep* **2021**, *11*, doi:10.1038/s41598-021-91093-7.

189. Mohan, S.; Singh, Y.; Verma, D.K.; Hasan, S.H. Synthesis of CuO Nanoparticles through Green Route Using Citrus Limon Juice and Its Application as Nanosorbent for Cr(VI) Remediation: Process Optimization with RSM and ANN-GA Based Model. *Process Safety and Environmental Protection* **2015**, *96*, 156–166, doi:10.1016/j.psep.2015.05.005.

190. Singh, H.P.; Sharma, S.; Sharma, S.K.; Sharma, R.K. Biogenic Synthesis of Metal Nanocatalysts Using Mimosa Pudica Leaves for Efficient Reduction of Aromatic Nitrocompounds. *RSC Adv* **2014**, *4*, 37816–37825, doi:10.1039/c4ra04233f.

191. Saif, S.; Adil, S.F.; Khan, M.; Hatshan, M.R.; Khan, M.; Bashir, F. Adsorption Studies of Arsenic(V) by Cuo Nanoparticles Synthesized by Phyllanthus Emblica Leaf-Extract-Fueled Solution Combustion Synthesis. *Sustainability (Switzerland)* **2021**, *13*, 1–14, doi:10.3390/su13042017.

192. Pakzad, K.; Alinezhad, H.; Nasrollahzadeh, M. Green Synthesis of Ni@Fe3O4 and CuO Nanoparticles Using Euphorbia Maculata Extract as Photocatalysts for the Degradation of Organic Pollutants under UV-Irradiation. *Ceram Int* **2019**, *45*, 17173–17182, doi:10.1016/j.ceramint.2019.05.272.
